# Insights into the performance of PREDICT tool in a large Mainland Chinese breast cancer cohort: a comparative analysis of versions 3.0 and 2.2

**DOI:** 10.1093/oncolo/oyae164

**Published:** 2024-06-29

**Authors:** Endong Chen, Chen Chen, Yingying Chen, Jie You, Chun Jin, Zhenxuan Huang, Jiayi Zhang, Qingxuan Wang, Yefeng Cai, Xiaoqu Hu, Quan Li

**Affiliations:** Department of Breast Surgery, The First Affiliated Hospital of Wenzhou Medical University, Wenzhou, Zhejiang, People’s Republic of China; The 1st School of Medicine, School of Information and Engineering, Wenzhou Medical University, Wenzhou, Zhejiang, People’s Republic of China; Department of Colorectal and Anal Surgery, The First Affiliated Hospital of Wenzhou Medical University, Wenzhou, Zhejiang, People’s Republic of China; Department of Thyroid Surgery, The First Affiliated Hospital of Wenzhou Medical University, Wenzhou, Zhejiang, People’s Republic of China; Department of Thyroid Surgery, The First Affiliated Hospital of Wenzhou Medical University, Wenzhou, Zhejiang, People’s Republic of China; The 1st School of Medicine, School of Information and Engineering, Wenzhou Medical University, Wenzhou, Zhejiang, People’s Republic of China; The 1st School of Medicine, School of Information and Engineering, Wenzhou Medical University, Wenzhou, Zhejiang, People’s Republic of China; Department of Thyroid Surgery, The First Affiliated Hospital of Wenzhou Medical University, Wenzhou, Zhejiang, People’s Republic of China; Department of Thyroid Surgery, The First Affiliated Hospital of Wenzhou Medical University, Wenzhou, Zhejiang, People’s Republic of China; Department of Breast Surgery, The First Affiliated Hospital of Wenzhou Medical University, Wenzhou, Zhejiang, People’s Republic of China; Department of Breast Surgery, The First Affiliated Hospital of Wenzhou Medical University, Wenzhou, Zhejiang, People’s Republic of China

**Keywords:** breast cancer, PREDICT, prediction model, chinese population, overall survival

## Abstract

**Background:**

PREDICT is a web-based tool for forecasting breast cancer outcomes. PREDICT version 3.0 was recently released. This study aimed to validate this tool for a large population in mainland China and compare v3.0 with v2.2.

**Methods:**

Women who underwent surgery for nonmetastatic primary invasive breast cancer between 2010 and 2020 from the First Affiliated Hospital of Wenzhou Medical University were selected. Predicted and observed 5-year overall survival (OS) for both v3.0 and v2.2 were compared. Discrimination was compared using receiver-operator curves and DeLong test. Calibration was evaluated using calibration plots and chi-squared test. A difference greater than 5% was deemed clinically relevant.

**Results:**

A total of 5424 patients were included, with median follow-up time of 58 months (IQR 38-89 months). Compared to v2.2, v3.0 did not show improved discriminatory accuracy for 5-year OS (AUC: 0.756 vs 0.771), same as ER-positive and ER-negative patients. However, calibration was significantly improved in v3.0, with predicted 5-year OS deviated from observed by −2.0% for the entire cohort, −2.9% for ER-positive and −0.0% for ER-negative patients, compared to −7.3%, −4.7% and −13.7% in v2.2. In v3.0, 5-year OS was underestimated by 9.0% for patients older than 75 years, and 5.8% for patients with micrometastases. Patients with distant metastases postdiagnosis was overestimated by 10.6%.

**Conclusions:**

PREDICT v3.0 reliably predicts 5-year OS for the majority of Chinese patients with breast cancer. PREDICT v3.0 significantly improved the predictive accuracy for ER-negative groups. Furthermore, caution is advised when interpreting 5-year OS for patients aged over 70, those with micrometastases or metastases postdiagnosis.

## Teaser

PREDICT is a web-based tool used to forecast breast cancer outcomes. This study aimed to validate this tool for a large population in mainland China and compare v3.0 with v2.2.

**Implications for Practice**

PREDICT is among the most popular prediction tools for breast cancer. This is the first extensive population-based study in mainland China, encompassing the full PREDICT target demographic. It systematically compares v3.0 with v2.2 and demonstrates the overall enhancements of PREDICT v3.0. For the first time, this analysis includes the HER2-low and postdiagnosis metastasis subgroups, and we suggest that integrating predictive factors for these metastases may enhance the model’s prognostic precision.

## Introduction

In the era of precision medicine, accurate prognosis prediction and informed systemic therapy decisions for patients with breast cancer are of paramount importance. Numerous predictive tools have been devised to estimate the survival rates of patients with breast cancer, aiding physicians in their treatment decisions.^1^

PREDICT is among the most prevalent prediction tools for breast cancer.^2^ Utilizing a series of clinicopathological indicators and treatment modalities, PREDICT offers prediction for a patient’s 5-, 10-, and 15-year survival as well as treatment benefits. In the past decade, PREDICT has seen continual updates aligned with advances in treatment research and clinicopathological indicators. This evolution is further refined through feedback from diverse validation datasets.^3-9^

The original PREDICT model (v1.0) was formulated by the Cambridge Breast Unit in 2010, drawing on data from the Eastern Cancer Registration and Information Centre. It used cancer registration and OS data for 5694 women diagnosed in East Anglia between 1999 and 2003, yielding estimates for both 5- and 10-year survival, along with quantified treatment advantages.^10^ In 2012, the model (v1.1) was updated to incorporate HER2/ERRB2 status, allowing for the calculation of trastuzumab treatment benefits.^11^ By 2014, the KI67 status was integrated as a prognostic factor in version 1.2, enhancing prediction accuracy for ER-positive patients.^12^ In subsequent versions (from v2.0 to v2.2), patient follow-up in the original cohort was extended to 10 years and the model was refitted for the first time. Version 2.0 considered the age at diagnosis and introduced smoothed hazard ratio functions for both tumor size and node status.^5^ Version 2.1 introduced bisphosphonates as a treatment option and added 15-year OS outcomes, while v2.2 further refined hormone therapy options. The training data for PREDICT v1.0 to v2.2 was based on patients diagnosed from 1993 to 2003, roughly 2 decades ago.

Given the significant evolution in early breast cancer survival rates (a 43% reduction in death rate from 1989 to 2020^13^) due to tremendous advancements in systemic treatment, it is evident that the model requires refinement to reflect the realities of the 2020s.

In July 2023, we were thrilled to witness the release of PREDICT v3.0. The PREDICT breast model was refitted using a national data set comprising patients diagnosed between 2000 and 2017. All hazard ratio estimates for the variables were refitted, and the year of diagnosis was factored into the prognosis to recalibrate the model for contemporary patients.^14^ In this version, estimations of the beneficial impact of radiotherapy on breast cancer mortality and the adverse effects of both chemotherapy and radiotherapy on other causes of mortality were included.

To date, PREDICT has been independently validated in cohorts from Canada,^3^ the Netherlands,^5,7,8^ the UK,^4,9^ and Malaysia (validated PREDICT v1.2).^6^ However, the performance of PREDICT in a large-scale dataset from mainland China remains unknown. This study aims to validate PREDICT v3.0 within a substantial population-based cohort of Chinese individuals. Separate analyses were performed to assess the model’s predictive performance within specific patient subgroups and to conduct a comprehensive analysis to validate both PREDICT versions, comparing v3.0 and v2.2 systematically.

## Methods

### Design

We obtained data on patient-, tumor-, and treatment-related characteristics from the First Affiliated Hospital of Wenzhou Medical University, China. Tumor staging adhered to the 8th edition of the tumor, node, and metastasis (TNM) classification system.^15^

The study’s endpoints was OS at 5 years, follow-up time was defined as the time from breast cancer diagnosis to last follow-up, death, or 5 years after diagnosis, whichever came first.^14^ Through the hospital’s records database and the *Wenzhou Health Service Platform*, we obtained the death registration information for patients. Analyses were censored on December 31, 2022 to allow for delay in reporting of vital status.

Information from the First Affiliated Hospital of WMU cohort contained age at diagnosis, menopausal status, ER, progesterone receptor (PR), HR (HR was deemed positive if at least 1% of invasive tumor cells exhibited immunostaining for ER or PR), HER2 status (categorized as positive, negative or low: HER2 IHC score of 1+ or 2+/ISH not amplified,^16^ HER2-low was treated as HER2-negative when entered into model). The dataset also included histology, histological grade, tumor size (mm), number of positive lymph nodes, AJCC stage, lymph or vascular invasion status, diagnosis year, metastasis (metastasis status after diagnosis, M1 was excluded), type of surgery (mastectomy or breast-conserving), lymph node procedure (SLNB, ALND, or SLNB + ALND), and type of adjuvant therapy (hormone therapy, chemotherapy [none, second generation, and third generation], targeted molecular therapy, and radiation therapy).

### Patients

Initially, 6741 women diagnosed with nonmetastatic primary invasive breast cancer between 2010 and 2020 were identified, all of whom underwent breast surgery. Patients confirmed diagnosis in other hospital and missing initial diagnosis information (*n* = 47), patients who had primary invasive malignant tumors before breast cancer diagnosis (*n* = 92), patients with bilateral breast invasive cancer (*n* = 147), patients who received neoadjuvant therapy (*n* = 350), patients aged outside the range of 25-85 (*n* = 31), patients with unknown missing ER status (*n* = 37), HER2 status (*n* = 6) or lymph node status (*n* = 255), node-negative patients with inadequate axillary node staging (<4 nodes sampled, *n* = 245) or patients with 20 or more positive lymph nodes (*n* = 103), and patients with tumor measuring 20 cm or larger (*n* = 4) were excluded. For missing data concerning tumor size (*n* = 318), tumor grade (*n* = 928), and KI67 status (*n* = 89), mean imputation was used, replacing the missing value with the variable’s mean.^12^ In total, data from 1135 patients underwent interpolation. This resulted in a final study cohort of 5424 patients, representing roughly 81% of the initially identified group.

Most patients presented with T1 (61%), N0 stage (58%), grade II (46%), ER-positive (69%), and HER2-negative disease (40%). The median age was 51 years (interquartile range: 45-59 years). The median follow-up of this population was 58 months (interquartile range 38-89).

### Statistical analysis

The observed 5-year survival rate was determined by calculating the proportion of patients who survived for 5 years relative to the total cohort, with the 95% CI derived using the Kaplan-Meier (KM) method.

The 5-year survival prediction was computed using the R code supplied by the PREDICT team (v3.0 and v2.2). Accounting for the treatments patients actually received, the predicted 5-year survival was ascertained by summing the survival prediction post-surgery and the estimated 5-year survival benefit from the treatment. The predicted proportions are not real proportions but reflect the sum of all predictions for each individual. All analyses were performed in R version 4.2.2 (The R Foundation, Vienna, Austria). A *P*-value threshold of .05 was defined as statistically significant.

Subsequently, we use a similar method applied in previous validations of PREDICT to assess the model’s discrimination and calibration.^17^ Model calibration was determined by plotting the averages of the observed against the predicted 5-year outcomes. A goodness-of-fit test was carried out by using a chi-squared test (*stats* package in R) based on the observed and predicted results (4 d.f.). We assumed that PREDICT accurately forecasted OS when the difference between predicted and observed outcomes remained within 5%; deviations beyond this margin were deemed to have clinical importance. A *P*-value below .05 was considered as statistically significant. Model discrimination was assessed by calculating the area under the receiver-operator characteristic curve (AUC) and corresponding 95% CIs for 5-year predicted OS and observed OS.^18^ (*pROC* package in R). Comparison between the AUC of v3.0 and v2.2 was made using DeLong test (*pROC* package in R).^11,19^

Finally, model reclassification was assessed through risk stratification. Patients were grouped into 3 categories based on their predicted 10-year breast cancer mortality: low-risk (0%-15%), medium-risk (15%-20%), and high-risk (>20%).^14^ This allowed for reclassification evaluation by comparing PREDICT v3.0 with v2.2. The KM plot illustrates the stratification effects among the risk groups for both predictive models.

## Results

### Model discrimination and calibration for 5-year OS

**Figure. 1.**
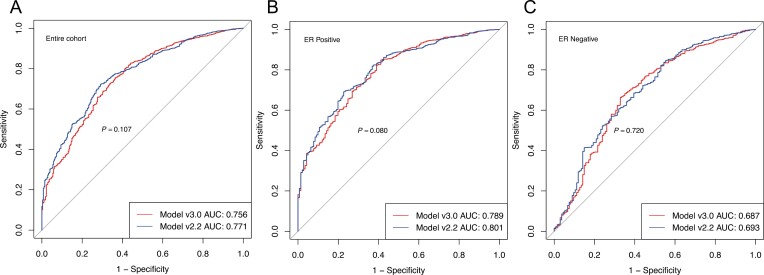
Discriminatory accuracy of 5-year overall survival for the entire cohort, ER-positive patients and ER-negative patients of PREDICT v3.0 and v2.2. (A) Entire cohort, (B) ER-positive group, and (C) ER-negative group. Abbreviation: ROC, receiver operating characteristic curve.

The PREDICT model discrimination by data set is shown in Figure 1. For the entire cohort, the discriminatory accuracy of v3.0 and v2.0 has no significant difference (AUC 0.756 vs 0.771, *P* = .107). The AUC values for PREDICT v3.0 and v2.2 were similar for both ER-positive and ER-negative disease as well (AUCs were 0.789 vs 0.801, *P* = .080; 0.687 vs 0.693, *P* = .720, respectively). Totally, for discrimination, PREDICT v3.0 performed slightly worse than v2.2.

In the entire WMU validation population, the predicted number of survivors after 5 years in the entire cohort was 5044.8 (93.0%, v3.0) and 4770.7 (88.0%, v2.2) compared to 5149 (94.9%) observed events. The difference was not significant in v3.0 (−2.0%, *P* = .963) while significant in v2.2 (−7.3%, *P* < .001).

**Figure. 2.**
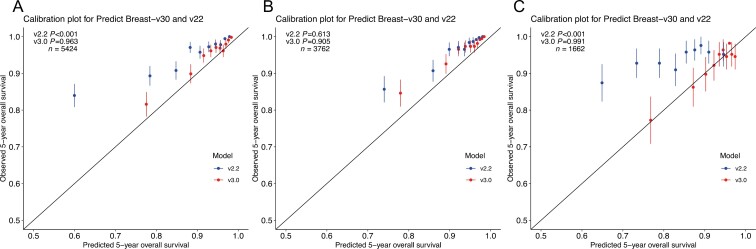
Observed and predicted 5-year overall survival for the entire cohort, ER-positive patients and ER-negative patients of PREDICT v3.0 and v2.2. (A) Entire cohort, (B) ER-positive group, and (C) ER-negative group.

In ER-positive patients, the difference between predicted and observed survivors was −2.9% (*P* = .905, v3.0) and −4.7% (*P* = .613, v2.2). In ER-negative patients, the difference between predicted and observed events was −0.0% (*P* = .991, v3.1) and −13.7% (*P* < .001, v2.2), which was not statistically significant in v3.0 but significant in v2.2 (Table 1). Figure 2 shows the predicted and observed 5-year OS by quintiles of the predicted survival.

**Table 1.** Baseline characteristic and observed and expected 5-year survival.

	Numbers and observed (%)		PREDICT Predict (%)		Difference (%)		*P*-value	
	n (%)	Observed	v3.0	v2.2	v3.0	v2.2	v3.0	v2.2
		(%, 95% CI)						
All patients	5424 (100.0)	5149 (94.9, 92.8-94.3)	5044.8 (93.0)	4770.7 (88.0)	−104.2 (−2.0)	−378.3 (−7.3)	.963	**<.001**
ER status								
Positive	3762 (69.4)	3621 (96.3, 94.3-95.9)	3516.1 (93.5)	3452.6 (91.8)	−104.9 (−2.9)	−168.4 (−4.7)	.905	.613
Negative	1662 (30.6)	1528 (91.9, 88.4-91.6)	1528.7 (92.0)	1318.1 (79.3)	0.7 (0.0)	−209.9 (−13.7)	.991	**<.001**
PR status								
Positive	3315 (61.1)	3212 (96.9, 95.2-96.7)	3113.7 (93.9)	3042.1 (91.8)	−98.3 (−3.1)	−169.9 (−5.3)	.834	.412
Negative	2089 (38.5)	1919 (91.9, 88.3-91.2)	1912.6 (91.6)	1711.2 (81.9)	−6.4 (−0.3)	−207.8 (−10.8)	.991	**<.001**
Unknown	20 (0.4)	18 (90.0, 73.0-100.0)	18.4 (92.0)	17.4 (87.0)	0.4 (2.2)	−0.6 (−3.3)	.993	.989
HER2 status								
Positive	1391 (25.6)	1310 (94.2, 90.7-94.0)	1288.3 (92.6)	1160.1 (83.4)	−21.7 (−1.7)	−149.9 (−11.4)	.999	**.001**
Negative	2147 (39.6)	2037 (94.9, 92.3-94.7)	1999.0 (93.1)	1912.5 (89.1)	−38.0 (−1.9)	−124.5 (−6.1)	.949	.213
Low	1886 (34.8)	1802 (95.5, 93.2-95.6)	1757.5 (93.2)	1698.2 (90.0)	−44.5 (−2.5)	−103.8 (−5.8)	.998	.612
Grade								
1	558 (10.3)	550 (98.6, 96.9-99.5)	533.5 (95.6)	532.7 (95.5)	−16.5 (−3.0)	−17.3 (−3.1)	.982	.999
2	2483 (45.8)	2356 (94.9, 92.4-94.6)	2320.1 (93.4)	2197.9 (88.5)	−35.9 (−1.5)	−158.1 (−6.7)	.981	**.025**
3	1455 (26.8)	1361 (93.5, 89.4-92.9)	1326.3 (91.2)	1192.4 (82.0)	−34.7 (−2.5)	−168.6 (−12.4)	.998	**.012**
Unknown	928 (17.1)	882 (95.0, 92.6-95.9)	864.9 (93.2)	847.7 (91.3)	−17.1 (−1.9)	−34.3 (−3.9)	.999	.990
Age at diagnosis								
<40	592 (10.9)	565 (95.4, 91.5-96.1)	565.0 (95.4)	537.9 (90.9)	0.0 (0.0)	−27.1 (−4.8)	.997	.813
40-49	1794 (33.1)	1737 (96.8, 94.8-96.9)	1715.9 (95.6)	1625.2 (90.6)	−21.1 (−1.2)	−111.8 (−6.4)	.997	.116
50-59	1709 (31.5)	1623 (95.0, 92.3-95.0)	1609.0 (94.1)	1504.4 (88.0)	−14.0 (−0.9)	−118.6 (−7.3)	.975	.102
60-69	990 (18.3)	936 (94.5, 91.4-95.0)	892.8 (90.2)	844.7 (85.3)	−43.2 (−4.6)	−91.3 (−9.8)	.998	.214
70-85	339 (6.2)	288 (85.0, 77.1-86.6)	262.2 (77.3)	258.5 (76.3)	−25.8 (−9.0)	−29.5 (−10.2)	.920	.820
Tumor size (mm)								
0-9	357 (6.6)	352 (98.6, 96.5-99.9)	344.1 (96.4)	339.0 (95.0)	−7.9 (−2.2)	−13.0 (−3.7)	.999	.992
10-19	1776 (32.7)	1714 (96.5, 94.5-96.7)	1681.4 (94.7)	1625.3 (91.5)	−32.6 (−1.9)	−88.7 (−5.2)	.987	.616
20-29	1689 (31.1)	1612 (95.4, 92.9-95.5)	1573.7 (93.2)	1487.6 (88.1)	−38.3 (−2.4)	−124.4 (−7.7)	.988	.128
30-49	1077 (19.9)	999 (92.8, 88.8-92.8)	971.7 (90.2)	883.6 (82.0)	−27.3 (−2.7)	−115.4 (−11.6)	.984	**.007**
50+	207 (3.8)	176 (85.0, 74.0-86.9)	179.1 (86.5)	154.2 (74.5)	3.1 (1.8)	−21.8 (−12.4)	.958	.647
Unknown	318 (5.9)	296 (93.1, 87.5-94.8)	294.8 (92.7)	280.9 (88.3)	−1.2 (−0.4)	−15.1 (−5.1)	.995	.946
Nodes positive								
0	3175 (58.5)	3101 (97.7, 96.3-97.7)	3013.4 (94.9)	2931.3 (92.3)	−87.6 (−2.8)	−169.7 (−5.5)	.826	.349
1	392 (7.2)	365 (93.1, 87.4-94.2)	365.0 (93.1)	349.4 (89.1)	0.0 (0.0)	−15.6 (−4.3)	.999	.836
2-4	980 (18.1)	924 (94.3, 91.0-94.7)	899.9 (91.8)	837.5 (85.5)	−24.1 (−2.6)	−86.5 (−9.4)	.953	.098
5-9	531 (9.8)	474 (89.3, 83.6-90.1)	474.5 (89.4)	414.2 (78.0)	0.5 (0.1)	−59.8 (−12.6)	.969	.057
10+	346 (6.4)	285 (82.4, 72.8-82.9)	292.0 (84.4)	238.3 (68.9)	7.0 (2.5)	−46.7 (−16.4)	.997	**.016**
Diagnosis year								
2010-2012	517 (9.5)	490 (94.8, 91.8-96.2)	481.9 (93.2)	457.3 (88.5)	−8.1 (−1.7)	−32.7 (−6.7)	.994	.383
2013-2016	1928 (35.5)	1773 (92.0, 90.3-92.9)	1789.8 (92.8)	1684.9 (87.4)	16.8 (0.9)	−88.1 (−5.0)	.924	.258
2017-2020	2979 (54.9)	2886 (96.9, 94.0-96.2)	2773.1 (93.1)	2628.5 (88.2)	−112.9 (−3.9)	−257.5 (−8.9)	.744	**<.001**
Hormone therapy								
Yes	3574 (65.9)	3443 (96.3, 94.5-96.1)	3349.7 (93.7)	3278.3 (91.7)	−93.3 (−2.7)	−164.7 (−4.8)	.928	.582
No	1850 (34.1)	1706 (92.2, 88.2-91.5)	1695.1 (91.6)	1492.5 (80.7)	−10.9 (−0.6)	−213.5 (−12.5)	.994	**<.001**
Generation chemotherapy								
No chemotherapy	1148 (21.2)	1084 (94.4, 90.6-94.3)	1044.6 (91.0)	1018.9 (88.8)	−39.4 (−3.6)	−65.1 (−6.0)	.805	.544
Generation 2	766 (14.1)	742 (96.9, 94.8-97.7)	720.9 (94.1)	695.7 (90.8)	−21.1 (−2.8)	−46.3 (−6.2)	.988	.573
Generation 3	3510 (64.7)	3323 (94.7, 92.3-94.2)	3279.3 (93.4)	3056.1 (87.1)	−43.7 (−1.3)	−266.9 (−8.0)	.995	**<.001**
Targeted molecular therapy								
Yes	945 (17.4)	902 (95.4, 91.6-95.5)	886.1 (93.8)	805.4 (85.2)	−15.9 (−1.8)	−96.6 (−10.7)	1.000	**.046**
No	4479 (82.6)	4247 (94.8, 92.7-94.3)	4158.7 (92.8)	3965.4 (88.5)	−88.3 (−2.1)	−281.6 (−6.6)	.955	**.003**
Radiation therapy								
Yes	1965 (36.2)	1840 (93.6, 90.8-93.5)	1847.2 (94.0)	1715.1 (87.3)	7.2 (0.4)	−124.9 (−6.8)	.974	.053
No	3459 (63.8)	3309 (95.7, 93.5-95.3)	3197.6 (92.4)	3055.6 (88.3)	−111.4 (−3.4)	−253.4 (−7.7)	.790	**.002**
Metastasis after diagnosis								
Yes	1038 (19.1)	864 (83.2, 78.3-83.5)	955.9 (92.1)	884.7 (85.2)	91.9 (10.6)	20.7 (2.4)	.399	.964
No	4386 (80.9)	4285 (97.7, 96.5-97.6)	4088.9 (93.2)	3886.0 (88.6)	−196.1 (−4.6)	−399.0 (−9.3)	.341	**<.001**

^*^The *P*-value was calculated by using a chi-squared test. *P*-values indicated in bold are considered as statistically significant (*P* < .05). >5% difference was considered as clinically relevant.

Abbreviations: N, total number; ER, estrogen receptor; PR, progesterone receptor; HER2, human epidermal growth factor receptor 2.

When analyzing PREDICT v3.0 in the predefined subgroups, 5-year OS was underestimated with 9.0% in patients older than 75 years. In patients with micrometastasis (pathologic N1mi), 5-year OS was also underestimated with 5.8%. Upon separate analysis of patients based on the presence or absence of distant metastases (considering only metastases postdiagnosis, as those with initial diagnosis metastases were excluded), the 5-year OS showed an overestimation by 10.6% in patients with metastases and an underestimation by 4.6% in those without (Table 1). Similar results were obtained by analyzing the presence or absence of metastases at specific sites (liver, bone, brain, and lung; Supplementary Table S1).

HER2-low breast cancer has recently emerged as a targetable subset of breast tumors, based on the evidence from clinical trials of novel anti-HER2 antibody-drug conjugates^20^). Approximately two-thirds of hormone receptor-positive and one-third of triple-negative tumors exhibit HER2-low expression (HER2 IHC score of 1+ or 2+/ISH not amplified^20^). It is interesting to explore whether HER2-negative and HER2-low patients show different prediction accuracy. For PREDICT v3.0, HER2-low subgroup discriminatory accuracy seems better than HER2-negative and HER2-positive (AUC: 0.802 vs 0.734, *P* = .045 and AUC: 0.802 vs 0.729, *P* = .049, Supplementary Figure S2A). The calibration plot shows no difference between HER2-low and negative (Supplementary Figure S2B). Besides, the accuracy of 5-year OS for HER2-positive patients has notably improved, shifting from −11.4% in v2.2 to −1.7% in v3.0 (refer to Table 1 and Supplementary Figure S2).

In all other predefined subgroups (entire cohort, PREDICT v3.0), no significant discrepancies were noted between predicted and observed events. While PREDICT v2.2 consistently underestimated the 5-year survival across most groups, the discrepancy was especially marked in the ER-negative group. The complete subgroup table of v3.0 and v2.2 of all indicators is available in Supplementary Table S1.

### Model reclassification

The Cambridge Breast Unit^14^ delineates 2 methods of risk stratification. The first approach categorizes women with breast cancer into 3 groups based on the anticipated 10-year benefit of adjuvant chemotherapy, measured by the absolute reduction in breast cancer-specific mortality risk; Women categorized as low-risk, with a forecasted 10-year benefit of 0%-3%, are typically advised against adjuvant chemotherapy. Conversely, high-risk women, with a projected benefit exceeding 5%, are generally recommended to undergo the treatment.^21^ For medium-risk women (3%-5%), recommendations are influenced by various factors, notably patient preferences. Alternative stratification approach is based on the predicted 10-year breast cancer mortality: low-risk (0%-15%), medium-risk (15%-20%), and high-risk (>20%). To investigate the correlation between anticipated mortality and observed outcomes, we chose the second stratification approach.

Using these risk categories, we evaluated reclassification by contrasting PREDICT v3.0 with v2.2. Out of 5424 breast cancer cases, 2061 (38%) women were categorized differently by PREDICT v2.2 and v3.0 (Table 2). In PREDICT v2.0, 82% (739/904) of the medium-risk patients were reclassified to low-risk in v3.0, while 55% (1279/2341) of the high-risk patients were shifted to either medium- or low-risk in v3.0. Compared to v3.0, PREDICT v2.2 notably overestimated the risk of death over a 10-year period.

**Table 2.** Reclassification of 5424 WMU breast cancer cases by PREDICT v3.0 into low-, medium- and high-risk compared to PREDICT v2.2 classification.

PREDICT v2.2	PREDICT v3.0			Total
	Low-risk	Medium-risk	High-risk	
Low-risk	2162	16	1	2179
Medium-risk	739	139	26	904
High-risk	799	480	1062	2341
Total	3700	635	1089	5424

**Figure. 3.**
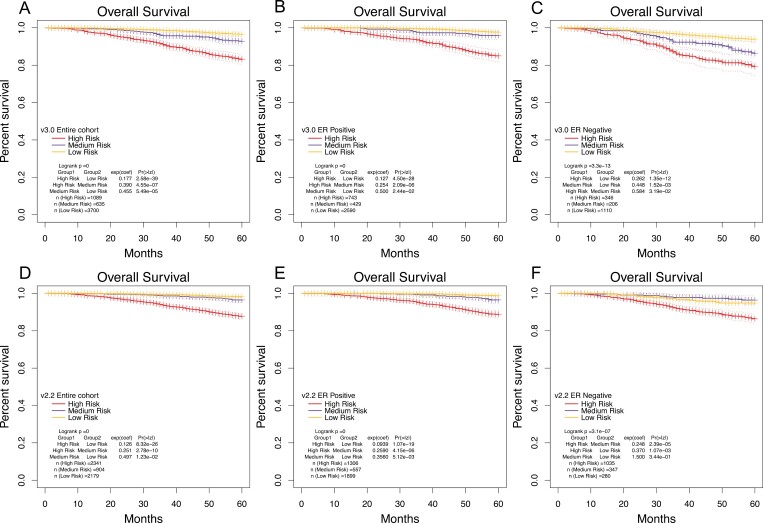
KM Survival curves according to the risk stratification. (A-C) Entire cohort, ER-positive and ER-negative of PREDICT v3.0 at 5years. (D-F) Entire cohort, ER-positive and ER-negative of PREDICT v2.2 at 5 years.

The KM survival curves post-risk stratification (Figure 3), reveal that PREDICT v3.0 outperforms v2.2 in differentiating between medium and low risks (for the entire cohort: medium-risk vs low-risk, *P* < .001 in v3.0 and *P* = .012 in v2.2). In ER-positive cases, the KM survival curve stratification is commendable for both PREDICT v3.0 and v2.2 (medium-risk vs low-risk, with *P*-values of .024 and <.01 respectively). However, for ER-negative patients, v3.0 shows a marked improvement over v2.2 (medium-risk vs low-risk, with *P*-values of .032 and .344, respectively).

## Discussion

In this study, we adopted inclusion criteria that align with those used by the PREDICT team^2,14^ and other prior validation studies.^5,7,8^ PREDICT v2.2 consistently underestimated 5-year OS in breast cancer, a finding aligned with initial research on v3.0.^14^ In PREDICT v3.0, updating the study cohorts and refitting the models significantly improved model calibration. We further validated the PREDICT’s 10-year OS prediction using patients diagnosed from 2010 to 2012, as detailed in Supplementary Table S2. The results align closely with the 5-year OS predictions. This progress is mainly attributed to the improvement of breast cancer prognosis. Further analyses of ER subgroups indicated that PREDICT v3.0 significantly improved predictive accuracy for both ER-negative and overall patients, with a minor improvement observed for ER-positive patients.

Our findings indicate that the 5-year OS prediction for patients above 75 years exhibited reduced accuracy, corresponding with the suboptimal precision observed in prior studies for the elderly population.^5,22^ In our dataset, the 5-year observed OS for patients with micrometastases (N1mi) was similar to those with N0. This might have contributed to the underestimation of 5-year survival for the micrometastases group.

While the AUC indicated no enhancement in model discrimination, it did not impact the model’s overarching efficacy, as model calibration, or goodness of fit, is typically deemed more crucial than discrimination and reflects the extent to which a model correctly estimates the absolute risk.^23^ For instance, in the previous study of Cambridge Unit, the AUC values in the model-fitting data exhibited a close resemblance between PREDICT v1.3 and v2.0 for ER-negative disease (0.724 and 0.726, *P* = .67), while for ER-positive disease, the AUC marginally rose from 0.791 to 0.796 (*P* = .028).^2^ While PREDICT v3.0 displayed only a marginal improvement in AUC compared to v2.2 (entire cohort in Eastern Region 0.824 vs 0.819, West Midlands 0.809 vs 0.806).^14^

This is the first study to conduct a subgroup analysis focused on distant metastases after initial diagnosis. Our findings indicate that PREDICT v3.0 considerably overestimates the 5-year OS for patients who developed distant metastases during follow-up. A plausible explanation is that even though patients with metastases were excluded from the model, a subset likely developed distant metastases as they continued their follow-up. This situation drastically worsened the prognosis for these patients, as a significant portion of the deaths occurred among those who developed metastases (out of the 5424 patients in our dataset, there were 275 total deaths in 5 years, and 174 of these were from the 1038 patients with metastases, accounting for 63% of all deaths). This is the reason the discrepancy is significant when analyzing patients with or without metastases separately during follow-up. Yet, when considered together, the prediction accuracy remains within a 5% range.

While PREDICT estimates survival probabilities for early-stage breast cancer, it does not mean that early-stage breast cancer will not develop distant metastases in the future. Owing to the inherent heterogeneity in breast cancer, distant metastasis risk varies across patient subtypes. Currently, PREDICT does not specify which patient subgroups are at elevated metastasis risk, leading to a significant overestimation of survival for those who eventually develop metastases during follow-up. To address this issue, we suggest that the potential risk of recurrence or metastasis be considered in the model. Future versions of the model could enhance predictive accuracy by either integrating variables that forecast tumor metastasis or by enhancing the weight of variables closely associated with recurrence or metastasis. Some new methods such as machine learning may be able to help establish risk predictions for distant metastases.^24^

Recent evidence from clinical trials on innovative anti-HER2 antibody-drug conjugates^16^ has highlighted HER2-low breast cancer as a potentially targetable tumor subset. A large proportion of breast cancer display HER2-low expression. Given its prevalence, understanding HER2-low is of significant clinical importance. Numerous studies investigate whether HER2-low can serve as a distinct type of breast cancer. Although the PREDICT model integrated HER2 status in 2012^11^ and the notion of HER2-low was introduced in the past 3 years, the distinction in predictive accuracy between HER2-negative and HER2-low patients within the model remains unknown. For the first time, we demonstrated that HER2-low and negative patients exhibit similar prediction accuracy. This finding suggests that HER2-low may not represent a unique survival subtype in breast cancer. Furthermore, our validation confirmed that PREDICT v3.0 offers enhanced accuracy for HER2-positive breast cancer, whereas v2.2 notably underestimates the 5-year OS. This improvement can likely be attributed to recent advancements in targeted treatments, which have substantially bettered the prognosis for patients with HER2-positive breast cancer.^25^

### Strengths and limitations

To the best of our knowledge, this study is the first population-based research within mainland China, encompassing the full PREDICT target demographic and offering the thorough validation of PREDICT v3.0 across diverse subgroups. Because of the large number of included populations, the model validation conclusions are relatively reliable. Additionally, our systematic comparison of v3.0 and v2.2, considering discrimination, calibration, risk stratification, and a thorough assessment of ER subgroups for each group, provides a holistic validation of both PREDICT versions. Moreover, our clinical dataset was notably comprehensive, encompassing detailed HER2 records enabling subclass validations for HER2-low patients. We have documented distant metastases occurring during the follow-up, and we suggest that integrating predictive factors for these metastases may bolster the model’s prognostic precision.

This study also has its limitations. First, the relatively short follow-up time precluded the validation of 10 and 15 years of prediction results. Second, lacking data on cause-specific mortality impedes our ability to discern if variations stem from breast cancer-specific deaths or other mortality causes. Third, the absence of detection mode data resulted in all patients being classified as clinically detected. The 5-year survival outcome might not fully capture the severity, given that symptomatic cancers often exhibit more adverse tumor traits than those detected through screening.^26^

## Conclusions

Within our extensive Chinese cohort, PREDICT v3.0 offers reliable OS predictions for the majority of Chinese patients with breast cancer. Although model discrimination did not see enhancement, PREDICT v3.0 notably surpasses v2.2 in nearly all subgroup analyses. Calibration in PREDICT v3.0 for ER-negative patients showed significant improvement, whereas only marginal enhancement was observed for ER-positive patients. PREDICT v3.0 exhibited a marked increase in prediction accuracy for HER2-positive patients, with negligible differences between HER2-low and negative groups. Additionally, caution is advised when interpreting 5-year OS for patients above 75, patients with micrometastases, and those diagnosed with metastases postinitial diagnosis. Model reclassification indicates a significant improvement in PREDICT’s risk stratification. In conclusion, PREDICT v3.0 has been well validated in the Chinese population. Given that PREDICT is free and does not necessitate genetic testing, it offers a significant advantage for both patients with breast cancer and clinicians in evaluating prognosis. We anticipate PREDICT becoming a useful and economic tool for breast cancer prognostic evaluations in China, benefiting a broader population.

## Supplementary Material

oyae164_suppl_Supplementary_Figure_S1

oyae164_suppl_Supplementary_Table_S1

oyae164_suppl_Supplementary_Table_S2

## Data Availability

The data underlying this article cannot be shared publicly due to reasons of confidentiality.
