# Supplementary material for: Insights into the performance of PREDICT tool in a large Mainland Chinese breast cancer cohort: a comparative analysis of versions 3.0 and 2.2
Source: Oncologist. 2024 Jun 29;29(8):e976–83. doi: 10.1093/oncolo/oyae164 (PMC11299932; doi:10.1093/oncolo/oyae164)
Supplement: oyae164_suppl_Supplementary_Figure_S1 [file oyae164_suppl_supplementary_figure_s1.pdf]

Fig S1

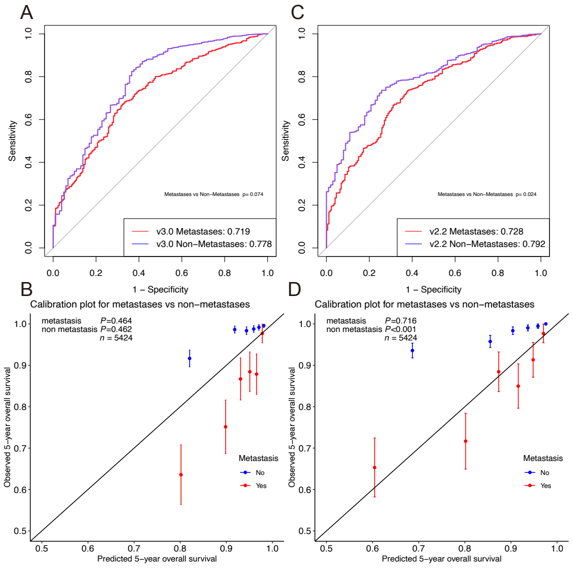

Fig. S1. **Discriminatory accuracy and calibration plot of 5-year overall survival for the different distant metastases status patients of PREDICT v3.0 and v2.2.** (A-B). PREDICT v3.0. (C-D). PREDICT v2.2.

Fig S2

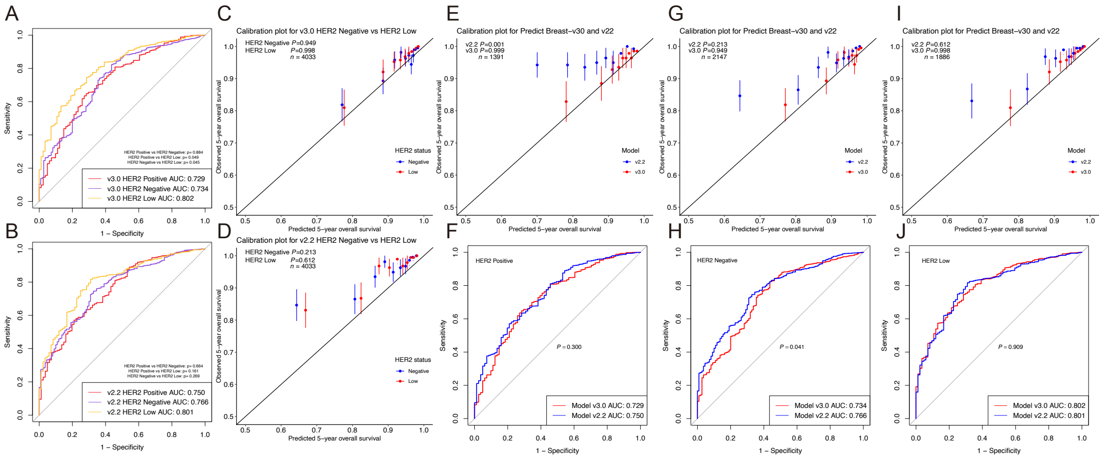

Fig. S2. **Discriminatory accuracy and calibration plot of 5-year overall survival for the different HER2 status of PREDICT v3.0 and v2.2.** (A-D) PREDICT v3.0 vs v2.0 (E-F) HER2-positive (G-H) HER2-low group (I-J) HER2-negative group.
