# Supplementary material for: Insights into the performance of PREDICT tool in a large Mainland Chinese breast cancer cohort: a comparative analysis of versions 3.0 and 2.2
Source: Oncologist. 2024 Jun 29;29(8):e976–83. doi: 10.1093/oncolo/oyae164 (PMC11299932; doi:10.1093/oncolo/oyae164)
Supplement: oyae164_suppl_Supplementary_Table_S1 [file oyae164_suppl_supplementary_table_s1.docx]

**Supplementary Table 1**

| **Table S1. Baseline characteristic and observed and expected 5-year survival.** | | | | | | | | |
| --- | --- | --- | --- | --- | --- | --- | --- | --- |
|  | **Numbers and Observed (%)** | | **PREDICT Predict (%)** | | **Difference (%)** | | **P-value** | |
|  | **n (%)** | **Observed** | **v3.0** | **v2.2** | **v3.0** | **v2.2** | **v3.0** | **v2.2** |
|  |  | **(%, 95% CI)** |  |  |  |  |  |  |
| **All Patients** | 5424 (100.0) | 5149 (94.9) | 5044.8 (93.0) | 4770.7 (88.0) | -104.2 (-2.0) | -378.3 (-7.3) | 0.963 | <0.001 |
|  |  | (92.8—94.3) |  |  |  |  |  |  |
| **ER Positive** | 3762 (69.4) | 3621 (96.3) | 3516.1 (93.5) | 3452.6 (91.8) | -104.9 (-2.9) | -168.4 (-4.7) | 0.905 | 0.613 |
|  |  | (94.3—95.9) |  |  |  |  |  |  |
| **ER Negative** | 1662 (30.6) | 1528 (91.9) | 1528.7 (92.0) | 1318.1 (79.3) | 0.7 (0.0) | -209.9 (-13.7) | 0.991 | <0.001 |
|  |  | (88.4—91.6) |  |  |  |  |  |  |
| **PR status** |  |  |  |  |  |  |  |  |
| Positive | 3315 (61.1) | 3212 (96.9) | 3113.7 (93.9) | 3042.1 (91.8) | -98.3 (-3.1) | -169.9 (-5.3) | 0.834 | 0.412 |
|  |  | (95.2—96.7) |  |  |  |  |  |  |
| Negative | 2089 (38.5) | 1919 (91.9) | 1912.6 (91.6) | 1711.2 (81.9) | -6.4 (-0.3) | -207.8 (-10.8) | 0.991 | <0.001 |
|  |  | (88.3—91.2) |  |  |  |  |  |  |
| Unknown | 20 (0.4) | 18 (90.0) | 18.4 (92.0) | 17.4 (87.0) | 0.4 (2.2) | -0.6 (-3.3) | 0.993 | 0.989 |
|  |  | (73.0—100.0) |  |  |  |  |  |  |
| **ER Positive** |  |  |  |  |  |  |  |  |
| Positive | 3195 (58.9) | 3101 (97.1) | 3001.8 (94.0) | 2945.7 (92.2) | -99.2 (-3.2) | -155.3 (-5.0) | 0.824 | 0.555 |
|  |  | (95.3—96.9) |  |  |  |  |  |  |
| Negative | 553 (10.2) | 508 (91.9) | 501.5 (90.7) | 494.4 (89.4) | -6.5 (-1.3) | -13.6 (-2.7) | 0.999 | 0.993 |
|  |  | (86.8—92.6) |  |  |  |  |  |  |
| Unknown | 14 (0.3) | 12 (85.7) | 12.9 (92.1) | 12.6 (90.0) | 0.9 (7.5) | 0.6 (5.0) | 0.971 | 0.972 |
|  |  | (62.1—100.0) |  |  |  |  |  |  |
| **ER Negative** |  |  |  |  |  |  |  |  |
| Positive | 120 (2.2) | 111 (92.5) | 112.0 (93.3) | 96.4 (80.3) | 1.0 (0.9) | -14.6 (-13.2) | 0.990 | 0.574 |
|  |  | (87.1—97.2) |  |  |  |  |  |  |
| Negative | 1536 (28.3) | 1411 (91.9) | 1411.1 (91.9) | 1216.8 (79.2) | 0.1 (0.0) | -194.2 (-13.8) | 0.994 | <0.001 |
|  |  | (88.1—91.5) |  |  |  |  |  |  |
| Unknown | 6 (0.1) | 6 (100.0) | 5.5 (91.7) | 4.9 (81.7) | -0.5 (-8.3) | -1.1 (-18.3) | 1.000 | 1.000 |
|  |  | (100.0—100.0) |  |  |  |  |  |  |
| **HER2 status** |  |  |  |  |  |  |  |  |
| Positive | 1391 (25.6) | 1310 (94.2) | 1288.3 (92.6) | 1160.1 (83.4) | -21.7 (-1.7) | -149.9 (-11.4) | 0.999 | 0.001 |
|  |  | (90.7—94.0) |  |  |  |  |  |  |
| Negative | 2147 (39.6) | 2037 (94.9) | 1999.0 (93.1) | 1912.5 (89.1) | -38.0 (-1.9) | -124.5 (-6.1) | 0.949 | 0.213 |
|  |  | (92.3—94.7) |  |  |  |  |  |  |
| Low | 1886 (34.8) | 1802 (95.5) | 1757.5 (93.2) | 1698.2 (90.0) | -44.5 (-2.5) | -103.8 (-5.8) | 0.998 | 0.612 |
|  |  | (93.2—95.6) |  |  |  |  |  |  |
| **ER Positive** |  |  |  |  |  |  |  |  |
| Positive | 660 (12.2) | 636 (96.4) | 618.3 (93.7) | 597.0 (90.5) | -17.7 (-2.8) | -39.0 (-6.1) | 0.997 | 0.826 |
|  |  | (92.8—96.9) |  |  |  |  |  |  |
| Negative | 1513 (27.9) | 1461 (96.6) | 1412.5 (93.4) | 1393.8 (92.1) | -48.5 (-3.3) | -67.2 (-4.6) | 0.905 | 0.860 |
|  |  | (94.4—96.8) |  |  |  |  |  |  |
| Low | 1589 (29.3) | 1524 (95.9) | 1485.3 (93.5) | 1461.8 (92.0) | -38.7 (-2.5) | -62.2 (-4.1) | 0.993 | 0.957 |
|  |  | (93.4—96.0) |  |  |  |  |  |  |
| **ER Negative** |  |  |  |  |  |  |  |  |
| Positive | 731 (13.5) | 674 (92.2) | 669.9 (91.6) | 563.1 (77.0) | -4.1 (-0.6) | -110.9 (-16.5) | 0.998 | 0.014 |
|  |  | (87.7—92.7) |  |  |  |  |  |  |
| Negative | 634 (11.7) | 576 (90.9) | 586.5 (92.5) | 518.7 (81.8) | 10.5 (1.8) | -57.3 (-9.9) | 0.960 | 0.166 |
|  |  | (85.9—91.5) |  |  |  |  |  |  |
| Low | 297 (5.5) | 278 (93.6) | 272.2 (91.6) | 236.4 (79.6) | -5.8 (-2.1) | -41.6 (-15.0) | 0.966 | 0.394 |
|  |  | (89.3—95.9) |  |  |  |  |  |  |
| **Grade** |  |  |  |  |  |  |  |  |
| 1 | 558 (10.3) | 550 (98.6) | 533.5 (95.6) | 532.7 (95.5) | -16.5 (-3.0) | -17.3 (-3.1) | 0.982 | 0.999 |
|  |  | (96.9—99.5) |  |  |  |  |  |  |
| 2 | 2483 (45.8) | 2356 (94.9) | 2320.1 (93.4) | 2197.9 (88.5) | -35.9 (-1.5) | -158.1 (-6.7) | 0.981 | 0.025 |
|  |  | (92.4—94.6) |  |  |  |  |  |  |
| 3 | 1455 (26.8) | 1361 (93.5) | 1326.3 (91.2) | 1192.4 (82.0) | -34.7 (-2.5) | -168.6 (-12.4) | 0.998 | 0.012 |
|  |  | (89.4—92.9) |  |  |  |  |  |  |
| Unknown | 928 (17.1) | 882 (95.0) | 864.9 (93.2) | 847.7 (91.3) | -17.1 (-1.9) | -34.3 (-3.9) | 0.999 | 0.990 |
|  |  | (92.6—95.9) |  |  |  |  |  |  |
| **ER Positive** |  |  |  |  |  |  |  |  |
| 1 | 529 (9.8) | 522 (98.7) | 506.1 (95.7) | 506.2 (95.7) | -15.9 (-3.0) | -15.8 (-3.0) | 0.974 | 0.999 |
|  |  | (97.3—99.6) |  |  |  |  |  |  |
| 2 | 1930 (35.6) | 1857 (96.2) | 1811.6 (93.9) | 1782.8 (92.4) | -45.4 (-2.4) | -74.2 (-4.0) | 0.992 | 0.960 |
|  |  | (93.8—96.1) |  |  |  |  |  |  |
| 3 | 678 (12.5) | 637 (94.0) | 615.0 (90.7) | 589.1 (86.9) | -22.0 (-3.5) | -47.9 (-7.5) | 0.993 | 0.660 |
|  |  | (88.6—93.9) |  |  |  |  |  |  |
| Unknown | 625 (11.5) | 605 (96.8) | 583.5 (93.4) | 574.5 (91.9) | -21.5 (-3.6) | -30.5 (-5.0) | 0.989 | 0.971 |
|  |  | (94.6—97.9) |  |  |  |  |  |  |
| **ER Negative** |  |  |  |  |  |  |  |  |
| 1 | 29 (0.5) | 28 (96.6) | 27.4 (94.5) | 26.4 (91.0) | -0.6 (-2.1) | -1.6 (-5.7) | 1.000 | 1.000 |
|  |  | (85.2—100.0) |  |  |  |  |  |  |
| 2 | 553 (10.2) | 499 (90.2) | 508.5 (92.0) | 415.1 (75.1) | 9.5 (1.9) | -83.9 (-16.8) | 0.985 | 0.069 |
|  |  | (85.4—91.4) |  |  |  |  |  |  |
| 3 | 777 (14.3) | 724 (93.2) | 711.3 (91.5) | 603.4 (77.7) | -12.7 (-1.8) | -120.6 (-16.7) | 0.999 | 0.070 |
|  |  | (88.7—93.5) |  |  |  |  |  |  |
| Unknown | 303 (5.6) | 277 (91.4) | 281.5 (92.9) | 273.2 (90.2) | 4.5 (1.6) | -3.8 (-1.4) | 0.998 | 0.986 |
|  |  | (86.5—93.8) |  |  |  |  |  |  |
| **Age at diagnosis** |  |  |  |  |  |  |  |  |
| <40 | 592 (10.9) | 565 (95.4) | 565.0 (95.4) | 537.9 (90.9) | 0.0 (0.0) | -27.1 (-4.8) | 0.997 | 0.813 |
|  |  | (91.5—96.1) |  |  |  |  |  |  |
| 40-49 | 1794 (33.1) | 1737 (96.8) | 1715.9 (95.6) | 1625.2 (90.6) | -21.1 (-1.2) | -111.8 (-6.4) | 0.997 | 0.116 |
|  |  | (94.8—96.9) |  |  |  |  |  |  |
| 50-59 | 1709 (31.5) | 1623 (95.0) | 1609.0 (94.1) | 1504.4 (88.0) | -14.0 (-0.9) | -118.6 (-7.3) | 0.975 | 0.102 |
|  |  | (92.3—95.0) |  |  |  |  |  |  |
| 60-69 | 990 (18.3) | 936 (94.5) | 892.8 (90.2) | 844.7 (85.3) | -43.2 (-4.6) | -91.3 (-9.8) | 0.998 | 0.214 |
|  |  | (91.4—95.0) |  |  |  |  |  |  |
| 70-85 | 339 (6.2) | 288 (85.0) | 262.2 (77.3) | 258.5 (76.3) | -25.8 (-9.0) | -29.5 (-10.2) | 0.920 | 0.820 |
|  |  | (77.1—86.6) |  |  |  |  |  |  |
| **ER Positive** |  |  |  |  |  |  |  |  |
| <40 | 435 (8.0) | 423 (97.2) | 418.1 (96.1) | 404.8 (93.1) | -4.9 (-1.2) | -18.2 (-4.3) | 0.999 | 0.916 |
|  |  | (93.9—98.3) |  |  |  |  |  |  |
| 40-49 | 1302 (24.0) | 1278 (98.2) | 1251.4 (96.1) | 1222.1 (93.9) | -26.6 (-2.1) | -55.9 (-4.4) | 0.995 | 0.855 |
|  |  | (96.3—98.4) |  |  |  |  |  |  |
| 50-59 | 1089 (20.1) | 1047 (96.1) | 1030.1 (94.6) | 1008.9 (92.6) | -16.9 (-1.6) | -38.1 (-3.6) | 1.000 | 0.975 |
|  |  | (93.3—96.4) |  |  |  |  |  |  |
| 60-69 | 683 (12.6) | 651 (95.3) | 619.3 (90.7) | 612.8 (89.7) | -31.7 (-4.9) | -38.2 (-5.9) | 0.994 | 0.996 |
|  |  | (91.9—96.1) |  |  |  |  |  |  |
| 70-85 | 253 (4.7) | 222 (87.7) | 197.2 (77.9) | 204.0 (80.6) | -24.8 (-11.2) | -18.0 (-8.1) | 0.880 | 0.868 |
|  |  | (80.5—90.3) |  |  |  |  |  |  |
| **ER Negative** |  |  |  |  |  |  |  |  |
| <40 | 157 (2.9) | 142 (90.4) | 146.8 (93.5) | 133.1 (84.8) | 4.8 (3.4) | -8.9 (-6.3) | 0.993 | 0.833 |
|  |  | (80.7—93.4) |  |  |  |  |  |  |
| 40-49 | 492 (9.1) | 459 (93.3) | 464.4 (94.4) | 403.1 (81.9) | 5.4 (1.2) | -55.9 (-12.2) | 0.997 | 0.089 |
|  |  | (88.8—94.4) |  |  |  |  |  |  |
| 50-59 | 620 (11.4) | 576 (92.9) | 578.8 (93.4) | 495.5 (79.9) | 2.8 (0.5) | -80.5 (-14.0) | 0.972 | 0.240 |
|  |  | (88.9—93.9) |  |  |  |  |  |  |
| 60-69 | 307 (5.7) | 285 (92.8) | 273.5 (89.1) | 231.9 (75.5) | -11.5 (-4.0) | -53.1 (-18.6) | 1.000 | 0.346 |
|  |  | (87.8—94.9) |  |  |  |  |  |  |
| 70-85 | 86 (1.6) | 66 (76.7) | 65.0 (75.6) | 54.5 (63.4) | -1.0 (-1.5) | -11.5 (-17.4) | 0.957 | 0.724 |
|  |  | (61.6—83.5) |  |  |  |  |  |  |
| **Tumor size(mm)** |  |  |  |  |  |  |  |  |
| 0-9 | 357 (6.6) | 352 (98.6) | 344.1 (96.4) | 339.0 (95.0) | -7.9 (-2.2) | -13.0 (-3.7) | 0.999 | 0.992 |
|  |  | (96.5—99.9) |  |  |  |  |  |  |
| 10-19 | 1776 (32.7) | 1714 (96.5) | 1681.4 (94.7) | 1625.3 (91.5) | -32.6 (-1.9) | -88.7 (-5.2) | 0.987 | 0.616 |
|  |  | (94.5—96.7) |  |  |  |  |  |  |
| 20-29 | 1689 (31.1) | 1612 (95.4) | 1573.7 (93.2) | 1487.6 (88.1) | -38.3 (-2.4) | -124.4 (-7.7) | 0.988 | 0.128 |
|  |  | (92.9—95.5) |  |  |  |  |  |  |
| 30-49 | 1077 (19.9) | 999 (92.8) | 971.7 (90.2) | 883.6 (82.0) | -27.3 (-2.7) | -115.4 (-11.6) | 0.984 | 0.007 |
|  |  | (88.8—92.8) |  |  |  |  |  |  |
| 50+ | 207 (3.8) | 176 (85.0) | 179.1 (86.5) | 154.2 (74.5) | 3.1 (1.8) | -21.8 (-12.4) | 0.958 | 0.647 |
|  |  | (74.0—86.9) |  |  |  |  |  |  |
| Unknown | 318 (5.9) | 296 (93.1) | 294.8 (92.7) | 280.9 (88.3) | -1.2 (-0.4) | -15.1 (-5.1) | 0.995 | 0.946 |
| **ER Positive** |  |  |  |  |  |  |  |  |
| 0-9 | 290 (5.3) | 286 (98.6) | 279.9 (96.5) | 279.8 (96.5) | -6.1 (-2.1) | -6.2 (-2.2) | 1.000 | 1.000 |
|  |  | (96.2—100.0) |  |  |  |  |  |  |
| 10-19 | 1332 (24.6) | 1305 (98.0) | 1264.9 (95.0) | 1253.3 (94.1) | -40.1 (-3.1) | -51.7 (-4.0) | 0.938 | 0.960 |
|  |  | (96.4—98.4) |  |  |  |  |  |  |
| 20-29 | 1132 (20.9) | 1093 (96.6) | 1057.4 (93.4) | 1037.5 (91.7) | -35.6 (-3.3) | -55.5 (-5.1) | 0.957 | 0.916 |
|  |  | (93.9—96.8) |  |  |  |  |  |  |
| 30-49 | 648 (11.9) | 606 (93.5) | 585.4 (90.3) | 565.6 (87.3) | -20.6 (-3.4) | -40.4 (-6.7) | 0.963 | 0.758 |
|  |  | (89.4—94.2) |  |  |  |  |  |  |
| 50+ | 131 (2.4) | 115 (87.8) | 114.2 (87.2) | 106.2 (81.1) | -0.8 (-0.7) | -8.8 (-7.7) | 0.996 | 0.664 |
|  |  | (75.0—91.0) |  |  |  |  |  |  |
| Unknown | 229 (4.2) | 216 (94.3) | 214.3 (93.6) | 210.2 (91.8) | -1.7 (-0.8) | -5.8 (-2.7) | 0.995 | 1.000 |
|  |  | (88.6—96.6) |  |  |  |  |  |  |
| **ER Negative** |  |  |  |  |  |  |  |  |
| 0-9 | 67 (1.2) | 66 (98.5) | 64.2 (95.8) | 59.1 (88.2) | -1.8 (-2.7) | -6.9 (-10.5) | 0.996 | 0.965 |
|  |  | (95.3—100.0) |  |  |  |  |  |  |
| 10-19 | 444 (8.2) | 409 (92.1) | 416.5 (93.8) | 372.1 (83.8) | 7.5 (1.8) | -36.9 (-9.0) | 0.994 | 0.740 |
|  |  | (87.1—93.4) |  |  |  |  |  |  |
| 20-29 | 557 (10.3) | 519 (93.2) | 516.2 (92.7) | 450.1 (80.8) | -2.8 (-0.5) | -68.9 (-13.3) | 0.999 | 0.133 |
|  |  | (89.3—94.4) |  |  |  |  |  |  |
| 30-49 | 429 (7.9) | 393 (91.6) | 386.3 (90.0) | 318.1 (74.1) | -6.7 (-1.7) | -74.9 (-19.1) | 1.000 | 0.032 |
|  |  | (85.9—92.7) |  |  |  |  |  |  |
| 50+ | 76 (1.4) | 61 (80.3) | 65.0 (85.5) | 48.1 (63.3) | 4.0 (6.6) | -12.9 (-21.1) | 0.983 | 0.886 |
|  |  | (66.1—87.7) |  |  |  |  |  |  |
| Unknown | 89 (1.6) | 80 (89.9) | 80.4 (90.3) | 70.7 (79.4) | 0.4 (0.5) | -9.3 (-11.6) | 0.974 | 0.186 |
|  |  | (79.4—95.5) |  |  |  |  |  |  |
| **Nodes positive** |  |  |  |  |  |  |  |  |
| 0 | 3175 (58.5) | 3101 (97.7) | 3013.4 (94.9) | 2931.3 (92.3) | -87.6 (-2.8) | -169.7 (-5.5) | 0.826 | 0.349 |
|  |  | (96.3—97.7) |  |  |  |  |  |  |
| 1 | 392 (7.2) | 365 (93.1) | 365.0 (93.1) | 349.4 (89.1) | 0.0 (0.0) | -15.6 (-4.3) | 0.999 | 0.836 |
|  |  | (87.4—94.2) |  |  |  |  |  |  |
| 2-4 | 980 (18.1) | 924 (94.3) | 899.9 (91.8) | 837.5 (85.5) | -24.1 (-2.6) | -86.5 (-9.4) | 0.953 | 0.098 |
|  |  | (91.0—94.7) |  |  |  |  |  |  |
| 5-9 | 531 (9.8) | 474 (89.3) | 474.5 (89.4) | 414.2 (78.0) | 0.5 (0.1) | -59.8 (-12.6) | 0.969 | 0.057 |
|  |  | (83.6—90.1) |  |  |  |  |  |  |
| 10+ | 346 (6.4) | 285 (82.4) | 292.0 (84.4) | 238.3 (68.9) | 7.0 (2.5) | -46.7 (-16.4) | 0.997 | 0.016 |
|  |  | (72.8—82.9) |  |  |  |  |  |  |
| **ER Positive** |  |  |  |  |  |  |  |  |
| 0 | 2191 (40.4) | 2159 (98.5) | 2086.2 (95.2) | 2076.0 (94.8) | -72.8 (-3.4) | -83.0 (-3.8) | 0.834 | 0.940 |
|  |  | (97.4—98.8) |  |  |  |  |  |  |
| 1 | 278 (5.1) | 265 (95.3) | 259.2 (93.2) | 256.1 (92.1) | -5.8 (-2.2) | -8.9 (-3.4) | 0.978 | 0.990 |
|  |  | (89.9—97.0) |  |  |  |  |  |  |
| 2-4 | 698 (12.9) | 665 (95.3) | 644.3 (92.3) | 629.7 (90.2) | -20.7 (-3.1) | -35.3 (-5.3) | 0.977 | 0.989 |
|  |  | (91.7—95.9) |  |  |  |  |  |  |
| 5-9 | 369 (6.8) | 337 (91.3) | 332.2 (90.0) | 314.1 (85.1) | -4.8 (-1.4) | -22.9 (-6.8) | 0.971 | 0.917 |
|  |  | (85.1—92.6) |  |  |  |  |  |  |
| 10+ | 226 (4.2) | 195 (86.3) | 194.3 (86.0) | 176.7 (78.2) | -0.7 (-0.4) | -18.3 (-9.4) | 0.957 | 0.968 |
|  |  | (77.5—88.7) |  |  |  |  |  |  |
| **ER Negative** |  |  |  |  |  |  |  |  |
| 0 | 984 (18.1) | 942 (95.7) | 927.2 (94.2) | 855.4 (86.9) | -14.8 (-1.6) | -86.6 (-9.2) | 0.964 | 0.734 |
|  |  | (92.7—96.1) |  |  |  |  |  |  |
| 1 | 114 (2.1) | 100 (87.7) | 105.8 (92.8) | 93.3 (81.8) | 5.8 (5.8) | -6.7 (-6.7) | 0.980 | 0.803 |
|  |  | (76.4—92.2) |  |  |  |  |  |  |
| 2-4 | 282 (5.2) | 259 (91.8) | 255.6 (90.6) | 207.7 (73.7) | -3.4 (-1.3) | -51.3 (-19.8) | 0.978 | 0.599 |
|  |  | (86.8—94.3) |  |  |  |  |  |  |
| 5-9 | 162 (3.0) | 137 (84.6) | 142.2 (87.8) | 100.1 (61.8) | 5.2 (3.8) | -36.9 (-26.9) | 0.998 | 0.702 |
|  |  | (75.9—88.7) |  |  |  |  |  |  |
| 10+ | 120 (2.2) | 90 (75.0) | 97.7 (81.4) | 61.6 (51.3) | 7.7 (8.6) | -28.4 (-31.6) | 0.864 | 0.011 |
|  |  | (57.5—77.7) |  |  |  |  |  |  |
| **Menopasus status** |  |  |  |  |  |  |  |  |
| Pre | 1201 (22.1) | 1166 (97.1) | 1150.8 (95.8) | 1102.1 (91.8) | -15.2 (-1.3) | -63.9 (-5.5) | 1.000 | 0.557 |
|  |  | (94.8—97.4) |  |  |  |  |  |  |
| Post | 1863 (34.3) | 1739 (93.3) | 1661.0 (89.2) | 1578.5 (84.7) | -78.0 (-4.5) | -160.5 (-9.2) | 0.978 | 0.031 |
|  |  | (90.2—93.1) |  |  |  |  |  |  |
| Unknown | 2360 (43.5) | 2244 (95.1) | 2232.9 (94.6) | 2090.1 (88.6) | -11.1 (-0.5) | -153.9 (-6.9) | 0.993 | 0.019 |
|  |  | (92.7—94.9) |  |  |  |  |  |  |
| **ER Positive** |  |  |  |  |  |  |  |  |
| Pre | 906 (16.7) | 890 (98.2) | 872.6 (96.3) | 851.9 (94.0) | -17.4 (-2.0) | -38.1 (-4.3) | 0.999 | 0.926 |
|  |  | (96.2—98.7) |  |  |  |  |  |  |
| Post | 1308 (24.1) | 1237 (94.6) | 1171.1 (89.5) | 1163.9 (89.0) | -65.9 (-5.3) | -73.1 (-5.9) | 0.950 | 0.976 |
|  |  | (91.6—94.8) |  |  |  |  |  |  |
| Unknown | 1548 (28.5) | 1494 (96.5) | 1472.4 (95.1) | 1436.8 (92.8) | -21.6 (-1.4) | -57.2 (-3.8) | 0.998 | 0.841 |
|  |  | (94.1—96.6) |  |  |  |  |  |  |
| **ER Negative** |  |  |  |  |  |  |  |  |
| Pre | 295 (5.4) | 276 (93.6) | 278.2 (94.3) | 250.3 (84.8) | 2.2 (0.8) | -25.7 (-9.3) | 0.999 | 0.721 |
|  |  | (87.7—95.3) |  |  |  |  |  |  |
| Post | 555 (10.2) | 502 (90.5) | 489.9 (88.3) | 414.5 (74.7) | -12.1 (-2.4) | -87.5 (-17.4) | 0.994 | 0.064 |
|  |  | (85.2—91.3) |  |  |  |  |  |  |
| Unknown | 812 (15.0) | 750 (92.4) | 760.6 (93.7) | 653.3 (80.5) | 10.6 (1.4) | -96.7 (-12.9) | 0.968 | 0.044 |
|  |  | (88.5—93.0) |  |  |  |  |  |  |
| **Pathologic T** |  |  |  |  |  |  |  |  |
| T1 | 3139 (57.9) | 3040 (96.8) | 2968.3 (94.6) | 2858.8 (91.1) | -71.7 (-2.4) | -181.2 (-6.0) | 0.925 | 0.110 |
|  |  | (95.2—96.8) |  |  |  |  |  |  |
| T2 | 1867 (34.4) | 1735 (92.9) | 1698.6 (91.0) | 1561.8 (83.7) | -36.4 (-2.1) | -173.2 (-10.0) | 0.986 | 0.003 |
|  |  | (89.4—92.4) |  |  |  |  |  |  |
| T3 | 85 (1.6) | 70 (82.4) | 73.0 (85.9) | 61.0 (71.8) | 3.0 (4.3) | -9.0 (-12.9) | 0.989 | 0.685 |
|  |  | (69.1—89.0) |  |  |  |  |  |  |
| T4 | 37 (0.7) | 29 (78.4) | 29.8 (80.5) | 26.7 (72.2) | 0.8 (2.8) | -2.3 (-7.9) | 0.989 | 0.839 |
|  |  | (57.3—90.8) |  |  |  |  |  |  |
| Unknown | 296 (5.5) | 275 (92.9) | 275.0 (92.9) | 262.4 (88.6) | 0.0 (0.0) | -12.6 (-4.6) | 0.995 | 0.975 |
|  |  | (87.4—94.9) |  |  |  |  |  |  |
| **ER Positive** |  |  |  |  |  |  |  |  |
| T1 | 2288 (42.2) | 2241 (97.9) | 2170.5 (94.9) | 2148.2 (93.9) | -70.5 (-3.1) | -92.8 (-4.1) | 0.861 | 0.873 |
|  |  | (96.5—98.0) |  |  |  |  |  |  |
| T2 | 1186 (21.9) | 1118 (94.3) | 1082.2 (91.2) | 1048.8 (88.4) | -35.8 (-3.2) | -69.2 (-6.2) | 0.960 | 0.577 |
|  |  | (90.8—94.3) |  |  |  |  |  |  |
| T3 | 52 (1.0) | 44 (84.6) | 44.8 (86.2) | 41.2 (79.2) | 0.8 (1.8) | -2.8 (-6.4) | 0.988 | 0.666 |
|  |  | (67.2—93.6) |  |  |  |  |  |  |
| T4 | 25 (0.5) | 20 (80.0) | 21.0 (84.0) | 20.3 (81.2) | 1.0 (5.0) | 0.3 (1.5) | 0.975 | 0.975 |
|  |  | (58.9—96.9) |  |  |  |  |  |  |
| Unknown | 211 (3.9) | 198 (93.8) | 197.6 (93.6) | 194.1 (92.0) | -0.4 (-0.2) | -3.9 (-2.0) | 0.994 | 1.000 |
|  |  | (87.9—96.4) |  |  |  |  |  |  |
| **ER Negative** |  |  |  |  |  |  |  |  |
| T1 | 851 (15.7) | 799 (93.9) | 797.8 (93.7) | 710.7 (83.5) | -1.2 (-0.2) | -88.3 (-11.1) | 0.990 | 0.145 |
|  |  | (90.6—94.6) |  |  |  |  |  |  |
| T2 | 681 (12.6) | 617 (90.6) | 616.5 (90.5) | 513.0 (75.3) | -0.5 (-0.1) | -104.0 (-16.9) | 0.992 | 0.024 |
|  |  | (85.1—90.8) |  |  |  |  |  |  |
| T3 | 33 (0.6) | 26 (78.8) | 28.2 (85.5) | 19.8 (60.0) | 2.2 (8.5) | -6.2 (-23.8) | 0.998 | 0.875 |
|  |  | (61.9—93.5) |  |  |  |  |  |  |
| T4 | 12 (0.2) | 9 (75.0) | 8.9 (74.2) | 6.4 (53.3) | -0.1 (-1.1) | -2.6 (-28.9) | 1.000 | 0.939 |
|  |  | (39.8—100.0) |  |  |  |  |  |  |
| Unknown | 85 (1.6) | 77 (90.6) | 77.4 (91.1) | 68.3 (80.4) | 0.4 (0.5) | -8.7 (-11.3) | 0.990 | 0.315 |
|  |  | (81.1—96.5) |  |  |  |  |  |  |
| **Pathologic N** |  |  |  |  |  |  |  |  |
| N0 | 3172 (58.5) | 3100 (97.7) | 3010.6 (94.9) | 2928.6 (92.3) | -89.4 (-2.9) | -171.4 (-5.5) | 0.816 | 0.339 |
|  |  | (96.4—97.7) |  |  |  |  |  |  |
| N1mi | 160 (2.9) | 156 (97.5) | 147.0 (91.9) | 137.7 (86.1) | -9.0 (-5.8) | -18.3 (-11.7) | 0.976 | 0.716 |
|  |  | (85.0—100.0) |  |  |  |  |  |  |
| N1 | 1078 (19.9) | 1009 (93.6) | 996.8 (92.5) | 938.2 (87.0) | -12.2 (-1.2) | -70.8 (-7.0) | 0.991 | 0.169 |
|  |  | (90.1—93.8) |  |  |  |  |  |  |
| N2 | 660 (12.2) | 598 (90.6) | 590.3 (89.4) | 520.6 (78.9) | -7.7 (-1.3) | -77.4 (-12.9) | 0.996 | 0.049 |
|  |  | (86.0—91.4) |  |  |  |  |  |  |
| N3 | 354 (6.5) | 286 (80.8) | 300.1 (84.8) | 245.6 (69.4) | 14.1 (4.9) | -40.4 (-14.1) | 0.991 | 0.006 |
|  |  | (71.2—81.4) |  |  |  |  |  |  |
| **ER Positive** |  |  |  |  |  |  |  |  |
| N0 | 2190 (40.4) | 2159 (98.6) | 2085.2 (95.2) | 2074.9 (94.7) | -73.8 (-3.4) | -84.1 (-3.9) | 0.840 | 0.941 |
|  |  | (97.5—98.8) |  |  |  |  |  |  |
| N1mi | 129 (2.4) | 125 (96.9) | 118.8 (92.1) | 115.3 (89.4) | -6.2 (-5.0) | -9.7 (-7.8) | 0.978 | 0.976 |
|  |  | (81.7—100.0) |  |  |  |  |  |  |
| N1 | 761 (14.0) | 724 (95.1) | 706.1 (92.8) | 693.5 (91.1) | -17.9 (-2.5) | -30.5 (-4.2) | 0.990 | 0.984 |
|  |  | (91.6—95.6) |  |  |  |  |  |  |
| N2 | 459 (8.5) | 425 (92.6) | 414.1 (90.2) | 394.0 (85.8) | -10.9 (-2.6) | -31.0 (-7.3) | 0.989 | 0.911 |
|  |  | (87.7—93.8) |  |  |  |  |  |  |
| N3 | 223 (4.1) | 188 (84.3) | 192.0 (86.1) | 174.9 (78.4) | 4.0 (2.1) | -13.1 (-7.0) | 0.947 | 0.911 |
|  |  | (75.2—86.8) |  |  |  |  |  |  |
| **ER Negative** |  |  |  |  |  |  |  |  |
| N0 | 982 (18.1) | 941 (95.8) | 925.4 (94.2) | 853.7 (86.9) | -15.6 (-1.7) | -87.3 (-9.3) | 0.959 | 0.709 |
|  |  | (92.9—96.2) |  |  |  |  |  |  |
| N1mi | 31 (0.6) | 31 (100.0) | 28.2 (91.0) | 22.5 (72.6) | -2.8 (-9.0) | -8.5 (-27.4) | 0.999 | 0.958 |
|  |  | (100.0—100.0) |  |  |  |  |  |  |
| N1 | 317 (5.8) | 285 (89.9) | 290.7 (91.7) | 244.7 (77.2) | 5.7 (2.0) | -40.3 (-14.1) | 0.965 | 0.418 |
|  |  | (84.0—92.0) |  |  |  |  |  |  |
| N2 | 201 (3.7) | 173 (86.1) | 176.2 (87.7) | 126.6 (63.0) | 3.2 (1.8) | -46.4 (-26.8) | 0.999 | 0.790 |
|  |  | (78.7—89.7) |  |  |  |  |  |  |
| N3 | 131 (2.4) | 98 (74.8) | 108.1 (82.5) | 70.7 (54.0) | 10.1 (10.3) | -27.3 (-27.9) | 0.683 | 0.007 |
|  |  | (58.6—77.5) |  |  |  |  |  |  |
| **AJCC stage** |  |  |  |  |  |  |  |  |
| I | 2154 (39.7) | 2117 (98.3) | 2056.8 (95.5) | 2010.9 (93.4) | -60.2 (-2.8) | -106.1 (-5.0) | 0.878 | 0.688 |
|  |  | (97.0—98.5) |  |  |  |  |  |  |
| II | 1978 (36.5) | 1890 (95.6) | 1838.9 (93.0) | 1745.3 (88.2) | -51.1 (-2.7) | -144.7 (-7.7) | 0.897 | 0.080 |
|  |  | (93.0—95.4) |  |  |  |  |  |  |
| III | 1056 (19.5) | 921 (87.2) | 927.1 (87.8) | 798.5 (75.6) | 6.1 (0.7) | -122.5 (-13.3) | 0.995 | <0.001 |
|  |  | (81.9—86.8) |  |  |  |  |  |  |
| Unknown | 236 (4.4) | 221 (93.6) | 222.0 (94.1) | 216.0 (91.5) | 1.0 (0.5) | -5.0 (-2.3) | 1.000 | 1.000 |
|  |  | (88.2—96.1) |  |  |  |  |  |  |
| **ER Positive** |  |  |  |  |  |  |  |  |
| I | 1573 (29.0) | 1557 (99.0) | 1505.5 (95.7) | 1499.0 (95.3) | -51.5 (-3.3) | -58.0 (-3.7) | 0.944 | 0.975 |
|  |  | (98.0—99.3) |  |  |  |  |  |  |
| II | 1309 (24.1) | 1264 (96.6) | 1219.7 (93.2) | 1202.4 (91.9) | -44.3 (-3.5) | -61.6 (-4.9) | 0.852 | 0.867 |
|  |  | (94.0—96.7) |  |  |  |  |  |  |
| III | 708 (13.1) | 636 (89.8) | 628.3 (88.7) | 590.1 (83.3) | -7.7 (-1.2) | -45.9 (-7.2) | 0.988 | 0.784 |
|  |  | (84.5—90.1) |  |  |  |  |  |  |
| Unknown | 172 (3.2) | 164 (95.3) | 162.7 (94.6) | 161.1 (93.7) | -1.3 (-0.8) | -2.9 (-1.8) | 0.999 | 0.996 |
|  |  | (90.1—98.3) |  |  |  |  |  |  |
| **ER Negative** |  |  |  |  |  |  |  |  |
| I | 581 (10.7) | 560 (96.4) | 551.2 (94.9) | 511.9 (88.1) | -8.8 (-1.6) | -48.1 (-8.6) | 0.971 | 0.811 |
|  |  | (93.3—97.3) |  |  |  |  |  |  |
| II | 669 (12.3) | 626 (93.6) | 619.3 (92.6) | 543.0 (81.2) | -6.7 (-1.1) | -83.0 (-13.3) | 0.993 | 0.419 |
|  |  | (89.5—94.3) |  |  |  |  |  |  |
| III | 348 (6.4) | 285 (81.9) | 298.9 (85.9) | 208.4 (59.9) | 13.9 (4.9) | -76.6 (-26.9) | 0.998 | 0.013 |
|  |  | (73.3—83.1) |  |  |  |  |  |  |
| Unknown | 64 (1.2) | 57 (89.1) | 59.3 (92.7) | 54.8 (85.6) | 2.3 (4.0) | -2.2 (-3.9) | 0.999 | 0.898 |
|  |  | (77.8—96.6) |  |  |  |  |  |  |
| **HR status** |  |  |  |  |  |  |  |  |
| Positive | 3882 (71.6) | 3732 (96.1) | 3628.1 (93.5) | 3549.0 (91.4) | -103.9 (-2.8) | -183.0 (-4.9) | 0.913 | 0.474 |
|  |  | (94.2—95.8) |  |  |  |  |  |  |
| Negative | 1542 (28.4) | 1417 (91.9) | 1416.7 (91.9) | 1221.7 (79.2) | -0.3 (-0.0) | -195.3 (-13.8) | 0.994 | <0.001 |
|  |  | (88.1—91.6) |  |  |  |  |  |  |
| **ER Positive** |  |  |  |  |  |  |  |  |
| Positive | 3762 (69.4) | 3621 (96.3) | 3516.1 (93.5) | 3452.6 (91.8) | -104.9 (-2.9) | -168.4 (-4.7) | 0.905 | 0.613 |
|  |  | (94.3—95.9) |  |  |  |  |  |  |
| **ER Negative** |  |  |  |  |  |  |  |  |
| Positive | 120 (2.2) | 111 (92.5) | 112.0 (93.3) | 96.4 (80.3) | 1.0 (0.9) | -14.6 (-13.2) | 0.990 | 0.574 |
|  |  | (87.1—97.2) |  |  |  |  |  |  |
| Negative | 1542 (28.4) | 1417 (91.9) | 1416.7 (91.9) | 1221.7 (79.2) | -0.3 (-0.0) | -195.3 (-13.8) | 0.994 | <0.001 |
|  |  | (88.1—91.6) |  |  |  |  |  |  |
| **Histology** |  |  |  |  |  |  |  |  |
| Ductal | 4523 (83.4) | 4276 (94.5) | 4202.5 (92.9) | 3955.0 (87.4) | -73.5 (-1.7) | -321.0 (-7.5) | 0.974 | <0.001 |
|  |  | (92.3—94.0) |  |  |  |  |  |  |
| Lobular | 137 (2.5) | 131 (95.6) | 128.9 (94.1) | 126.0 (92.0) | -2.1 (-1.6) | -5.0 (-3.8) | 0.993 | 0.997 |
|  |  | (90.0—98.9) |  |  |  |  |  |  |
| Mixed ductal/lobular | 133 (2.5) | 128 (96.2) | 124.4 (93.5) | 119.1 (89.5) | -3.6 (-2.8) | -8.9 (-7.0) | 0.999 | 0.957 |
|  |  | (90.6—99.4) |  |  |  |  |  |  |
| Others | 631 (11.6) | 614 (97.3) | 589.1 (93.4) | 570.7 (90.4) | -24.9 (-4.1) | -43.3 (-7.1) | 0.981 | 0.792 |
|  |  | (95.2—98.3) |  |  |  |  |  |  |
| **ER Positive** |  |  |  |  |  |  |  |  |
| Ductal | 3090 (57.0) | 2966 (96.0) | 2886.7 (93.4) | 2831.5 (91.6) | -79.3 (-2.7) | -134.5 (-4.5) | 0.934 | 0.650 |
|  |  | (93.9—95.7) |  |  |  |  |  |  |
| Lobular | 109 (2.0) | 103 (94.5) | 102.6 (94.1) | 101.1 (92.8) | -0.4 (-0.4) | -1.9 (-1.8) | 0.980 | 0.983 |
|  |  | (87.7—98.7) |  |  |  |  |  |  |
| Mixed ductal/lobular | 112 (2.1) | 109 (97.3) | 105.1 (93.8) | 103.3 (92.2) | -3.9 (-3.6) | -5.7 (-5.2) | 0.989 | 0.997 |
|  |  | (92.4—100.0) |  |  |  |  |  |  |
| Others | 451 (8.3) | 443 (98.2) | 421.7 (93.5) | 416.7 (92.4) | -21.3 (-4.8) | -26.3 (-5.9) | 0.979 | 0.972 |
|  |  | (96.4—99.4) |  |  |  |  |  |  |
| **ER Negative** |  |  |  |  |  |  |  |  |
| Ductal | 1433 (26.4) | 1310 (91.4) | 1315.7 (91.8) | 1123.4 (78.4) | 5.7 (0.4) | -186.6 (-14.2) | 0.983 | <0.001 |
|  |  | (87.8—91.3) |  |  |  |  |  |  |
| Lobular | 28 (0.5) | 28 (100.0) | 26.3 (93.9) | 24.9 (88.9) | -1.7 (-6.1) | -3.1 (-11.1) | 1.000 | 0.997 |
|  |  | (100.0—100.0) |  |  |  |  |  |  |
| Mixed ductal/lobular | 21 (0.4) | 19 (90.5) | 19.3 (91.9) | 15.8 (75.2) | 0.3 (1.6) | -3.2 (-16.8) | 0.986 | 0.930 |
|  |  | (65.7—100.0) |  |  |  |  |  |  |
| Others | 180 (3.3) | 171 (95.0) | 167.4 (93.0) | 154.0 (85.6) | -3.6 (-2.1) | -17.0 (-9.9) | 1.000 | 0.847 |
|  |  | (89.8—97.9) |  |  |  |  |  |  |
| **Diagnosis year** |  |  |  |  |  |  |  |  |
| 2010-2012 | 517 (9.5) | 490 (94.8) | 481.9 (93.2) | 457.3 (88.5) | -8.1 (-1.7) | -32.7 (-6.7) | 0.994 | 0.383 |
|  |  | (91.8—96.2) |  |  |  |  |  |  |
| 2013-2016 | 1928 (35.5) | 1773 (92.0) | 1789.8 (92.8) | 1684.9 (87.4) | 16.8 (0.9) | -88.1 (-5.0) | 0.924 | 0.258 |
|  |  | (90.3—92.9) |  |  |  |  |  |  |
| 2017-2020 | 2979 (54.9) | 2886 (96.9) | 2773.1 (93.1) | 2628.5 (88.2) | -112.9 (-3.9) | -257.5 (-8.9) | 0.744 | <0.001 |
|  |  | (94.0—96.2) |  |  |  |  |  |  |
| **ER Positive** |  |  |  |  |  |  |  |  |
| 2010-2012 | 340 (6.3) | 325 (95.6) | 319.6 (94.0) | 314.1 (92.4) | -5.4 (-1.7) | -10.9 (-3.4) | 0.998 | 0.988 |
|  |  | (92.5—97.5) |  |  |  |  |  |  |
| 2013-2016 | 1291 (23.8) | 1216 (94.2) | 1206.1 (93.4) | 1184.2 (91.7) | -9.9 (-0.8) | -31.8 (-2.6) | 0.997 | 0.999 |
|  |  | (92.6—95.3) |  |  |  |  |  |  |
| 2017-2020 | 2131 (39.3) | 2080 (97.6) | 1990.4 (93.4) | 1954.4 (91.7) | -89.6 (-4.3) | -125.6 (-6.0) | 0.704 | 0.468 |
|  |  | (95.0—97.3) |  |  |  |  |  |  |
| **ER Negative** |  |  |  |  |  |  |  |  |
| 2010-2012 | 177 (3.3) | 165 (93.2) | 162.3 (91.7) | 143.2 (80.9) | -2.7 (-1.6) | -21.8 (-13.2) | 0.961 | 0.091 |
|  |  | (87.9—96.5) |  |  |  |  |  |  |
| 2013-2016 | 637 (11.7) | 557 (87.4) | 583.7 (91.6) | 500.7 (78.6) | 26.7 (4.8) | -56.3 (-10.1) | 0.922 | 0.204 |
|  |  | (84.1—89.5) |  |  |  |  |  |  |
| 2017-2020 | 848 (15.6) | 806 (95.0) | 782.7 (92.3) | 674.2 (79.5) | -23.3 (-2.9) | -131.8 (-16.4) | 0.981 | 0.025 |
|  |  | (90.1—95.0) |  |  |  |  |  |  |
| **Lymph or vascular invasion** | |  |  |  |  |  |  |  |
| Yes | 1134 (20.9) | 1037 (91.4) | 1031.9 (91.0) | 938.7 (82.8) | -5.1 (-0.5) | -98.3 (-9.5) | 0.979 | 0.002 |
|  |  | (86.7—91.0) |  |  |  |  |  |  |
| No | 2723 (50.2) | 2651 (97.4) | 2554.7 (93.8) | 2443.7 (89.7) | -96.3 (-3.6) | -207.3 (-7.8) | 0.742 | 0.014 |
|  |  | (95.5—97.2) |  |  |  |  |  |  |
| Unknown | 1567 (28.9) | 1461 (93.2) | 1458.2 (93.1) | 1388.4 (88.6) | -2.8 (-0.2) | -72.6 (-5.0) | 0.983 | 0.571 |
|  |  | (91.2—93.9) |  |  |  |  |  |  |
| **ER Positive** |  |  |  |  |  |  |  |  |
| Yes | 799 (14.7) | 748 (93.6) | 731.5 (91.6) | 704.7 (88.2) | -16.5 (-2.2) | -43.3 (-5.8) | 0.912 | 0.603 |
|  |  | (88.9—93.6) |  |  |  |  |  |  |
| No | 1942 (35.8) | 1903 (98.0) | 1829.0 (94.2) | 1805.6 (93.0) | -74.0 (-3.9) | -97.4 (-5.1) | 0.834 | 0.738 |
|  |  | (96.1—98.0) |  |  |  |  |  |  |
| Unknown | 1021 (18.8) | 970 (95.0) | 955.7 (93.6) | 942.2 (92.3) | -14.3 (-1.5) | -27.8 (-2.9) | 0.996 | 1.000 |
|  |  | (93.1—96.0) |  |  |  |  |  |  |
| **ER Negative** |  |  |  |  |  |  |  |  |
| Yes | 335 (6.2) | 289 (86.3) | 300.4 (89.7) | 234.0 (69.9) | 11.4 (3.9) | -55.0 (-19.0) | 0.995 | 0.014 |
|  |  | (78.9—87.9) |  |  |  |  |  |  |
| No | 781 (14.4) | 748 (95.8) | 725.7 (92.9) | 638.0 (81.7) | -22.3 (-3.0) | -110.0 (-14.7) | 0.976 | 0.080 |
|  |  | (92.5—96.4) |  |  |  |  |  |  |
| Unknown | 546 (10.1) | 491 (89.9) | 502.5 (92.0) | 446.1 (81.7) | 11.5 (2.3) | -44.9 (-9.1) | 0.955 | 0.316 |
|  |  | (86.0—91.6) |  |  |  |  |  |  |
| **Anatomic neoplasm** | |  |  |  |  |  |  |  |
| Left | 2789 (51.4) | 2655 (95.2) | 2595.9 (93.1) | 2452.0 (87.9) | -59.1 (-2.2) | -203.0 (-7.6) | 0.971 | 0.007 |
|  |  | (93.0—95.0) |  |  |  |  |  |  |
| Right | 2635 (48.6) | 2494 (94.6) | 2448.9 (92.9) | 2318.7 (88.0) | -45.1 (-1.8) | -175.3 (-7.0) | 0.997 | 0.012 |
|  |  | (92.0—94.2) |  |  |  |  |  |  |
| **ER Positive** |  |  |  |  |  |  |  |  |
| Left | 1908 (35.2) | 1837 (96.3) | 1783.9 (93.5) | 1750.4 (91.7) | -53.1 (-2.9) | -86.6 (-4.7) | 0.957 | 0.825 |
|  |  | (94.0—96.3) |  |  |  |  |  |  |
| Right | 1854 (34.2) | 1784 (96.2) | 1732.2 (93.4) | 1702.2 (91.8) | -51.8 (-2.9) | -81.8 (-4.6) | 0.978 | 0.877 |
|  |  | (93.9—96.2) |  |  |  |  |  |  |
| **ER Negative** |  |  |  |  |  |  |  |  |
| Left | 881 (16.2) | 818 (92.8) | 812.0 (92.2) | 701.5 (79.6) | -6.0 (-0.7) | -116.5 (-14.2) | 0.995 | 0.031 |
|  |  | (89.3—93.4) |  |  |  |  |  |  |
| Right | 781 (14.4) | 710 (90.9) | 716.7 (91.8) | 616.6 (79.0) | 6.7 (0.9) | -93.4 (-13.2) | 0.993 | 0.024 |
|  |  | (86.0—91.1) |  |  |  |  |  |  |
| **Metastasis after diagnosis** | |  |  |  |  |  |  |  |
| Yes | 1038 (19.1) | 864 (83.2) | 955.9 (92.1) | 884.7 (85.2) | 91.9 (10.6) | 20.7 (2.4) | 0.399 | 0.964 |
|  |  | (78.3—83.5) |  |  |  |  |  |  |
| No | 4386 (80.9) | 4285 (97.7) | 4088.9 (93.2) | 3886.0 (88.6) | -196.1 (-4.6) | -399.0 (-9.3) | 0.341 | <0.001 |
|  |  | (96.5—97.6) |  |  |  |  |  |  |
| **ER Positive** |  |  |  |  |  |  |  |  |
| Yes | 661 (12.2) | 577 (87.3) | 612.8 (92.7) | 596.7 (90.3) | 35.8 (6.2) | 19.7 (3.4) | 0.843 | 0.963 |
|  |  | (82.5—88.3) |  |  |  |  |  |  |
| No | 3101 (57.2) | 3044 (98.2) | 2903.3 (93.6) | 2855.9 (92.1) | -140.7 (-4.6) | -188.1 (-6.2) | 0.505 | 0.211 |
|  |  | (97.0—98.2) |  |  |  |  |  |  |
| **ER Negative** |  |  |  |  |  |  |  |  |
| Yes | 377 (7.0) | 287 (76.1) | 343.0 (91.0) | 287.9 (76.4) | 56.0 (19.5) | 0.9 (0.3) | 0.640 | 0.691 |
|  |  | (68.2—77.9) |  |  |  |  |  |  |
| No | 1285 (23.7) | 1241 (96.6) | 1185.6 (92.3) | 1030.2 (80.2) | -55.4 (-4.5) | -210.8 (-17.0) | 0.836 | <0.001 |
|  |  | (94.5—97.0) |  |  |  |  |  |  |
| **Liver Metastasis after diagnosis** | |  |  |  |  |  |  |  |
| Yes | 198 (3.7) | 133 (67.2) | 182.3 (92.1) | 166.6 (84.1) | 49.3 (37.1) | 33.6 (25.3) | 0.360 | 0.950 |
|  |  | (57.5—71.6) |  |  |  |  |  |  |
| No | 5226 (96.3) | 5016 (96.0) | 4862.5 (93.0) | 4604.1 (88.1) | -153.5 (-3.1) | -411.9 (-8.2) | 0.808 | <0.001 |
|  |  | (94.2—95.6) |  |  |  |  |  |  |
| **ER Positive** |  |  |  |  |  |  |  |  |
| Yes | 126 (2.3) | 90 (71.4) | 116.3 (92.3) | 112.2 (89.0) | 26.3 (29.2) | 22.2 (24.7) | 0.559 | 0.796 |
|  |  | (60.3—77.6) |  |  |  |  |  |  |
| No | 3636 (67.0) | 3531 (97.1) | 3399.9 (93.5) | 3340.4 (91.9) | -131.1 (-3.7) | -190.6 (-5.4) | 0.747 | 0.393 |
|  |  | (95.5—97.0) |  |  |  |  |  |  |
| **ER Negative** |  |  |  |  |  |  |  |  |
| Yes | 72 (1.3) | 43 (59.7) | 66.0 (91.7) | 54.4 (75.6) | 23.0 (53.5) | 11.4 (26.5) | 0.384 | 0.965 |
|  |  | (46.3—70.4) |  |  |  |  |  |  |
| No | 1590 (29.3) | 1485 (93.4) | 1462.7 (92.0) | 1263.7 (79.5) | -22.3 (-1.5) | -221.3 (-14.9) | 0.978 | <0.001 |
|  |  | (90.3—93.3) |  |  |  |  |  |  |
| **Bone Metastasis after diagnosis** | |  |  |  |  |  |  |  |
| Yes | 558 (10.3) | 450 (80.6) | 514.2 (92.2) | 475.5 (85.2) | 64.2 (14.3) | 25.5 (5.7) | 0.625 | 0.836 |
|  |  | (75.1—82.3) |  |  |  |  |  |  |
| No | 4866 (89.7) | 4699 (96.6) | 4530.6 (93.1) | 4295.3 (88.3) | -168.4 (-3.6) | -403.7 (-8.6) | 0.742 | <0.001 |
|  |  | (95.0—96.3) |  |  |  |  |  |  |
| **ER Positive** |  |  |  |  |  |  |  |  |
| Yes | 364 (6.7) | 305 (83.8) | 336.7 (92.5) | 326.4 (89.7) | 31.7 (10.4) | 21.4 (7.0) | 0.873 | 0.945 |
|  |  | (77.9—86.3) |  |  |  |  |  |  |
| No | 3398 (62.6) | 3316 (97.6) | 3179.4 (93.6) | 3126.2 (92.0) | -136.6 (-4.1) | -189.8 (-5.7) | 0.707 | 0.333 |
|  |  | (96.2—97.6) |  |  |  |  |  |  |
| **ER Negative** |  |  |  |  |  |  |  |  |
| Yes | 194 (3.6) | 145 (74.7) | 177.5 (91.5) | 149.1 (76.9) | 32.5 (22.4) | 4.1 (2.8) | 0.683 | 0.723 |
|  |  | (65.9—79.3) |  |  |  |  |  |  |
| No | 1468 (27.1) | 1383 (94.2) | 1351.2 (92.0) | 1169.1 (79.6) | -31.8 (-2.3) | -213.9 (-15.5) | 0.977 | <0.001 |
|  |  | (91.1—94.2) |  |  |  |  |  |  |
| **Brain Metastasis after diagnosis** | |  |  |  |  |  |  |  |
| Yes | 225 (4.1) | 181 (80.4) | 208.4 (92.6) | 190.9 (84.8) | 27.4 (15.1) | 9.9 (5.5) | 0.640 | 0.962 |
|  |  | (72.1—83.8) |  |  |  |  |  |  |
| No | 5199 (95.9) | 4968 (95.6) | 4836.4 (93.0) | 4579.8 (88.1) | -131.6 (-2.6) | -388.2 (-7.8) | 0.889 | <0.001 |
|  |  | (93.6—95.1) |  |  |  |  |  |  |
| **ER Positive** |  |  |  |  |  |  |  |  |
| Yes | 130 (2.4) | 112 (86.2) | 121.3 (93.3) | 117.4 (90.3) | 9.3 (8.3) | 5.4 (4.8) | 0.919 | 0.978 |
|  |  | (77.7—91.2) |  |  |  |  |  |  |
| No | 3632 (67.0) | 3509 (96.6) | 3394.8 (93.5) | 3335.2 (91.8) | -114.2 (-3.3) | -173.8 (-5.0) | 0.844 | 0.498 |
|  |  | (94.8—96.3) |  |  |  |  |  |  |
| **ER Negative** |  |  |  |  |  |  |  |  |
| Yes | 95 (1.8) | 69 (72.6) | 87.0 (91.6) | 73.5 (77.4) | 18.0 (26.1) | 4.5 (6.5) | 0.687 | 0.630 |
|  |  | (59.2—79.6) |  |  |  |  |  |  |
| No | 1567 (28.9) | 1459 (93.1) | 1441.6 (92.0) | 1244.6 (79.4) | -17.4 (-1.2) | -214.4 (-14.7) | 0.986 | <0.001 |
|  |  | (89.8—93.0) |  |  |  |  |  |  |
| **Lung Metastasis after diagnosis** | |  |  |  |  |  |  |  |
| Yes | 214 (3.9) | 143 (66.8) | 194.8 (91.0) | 176.3 (82.4) | 51.8 (36.2) | 33.3 (23.3) | 0.360 | 0.955 |
|  |  | (57.1—70.8) |  |  |  |  |  |  |
| No | 5210 (96.1) | 5006 (96.1) | 4850.0 (93.1) | 4594.4 (88.2) | -156.0 (-3.1) | -411.6 (-8.2) | 0.776 | <0.001 |
|  |  | (94.3—95.7) |  |  |  |  |  |  |
| **ER Positive** |  |  |  |  |  |  |  |  |
| Yes | 111 (2.0) | 86 (77.5) | 102.1 (92.0) | 98.4 (88.6) | 16.1 (18.7) | 12.4 (14.4) | 0.946 | 0.996 |
|  |  | (67.3—84.2) |  |  |  |  |  |  |
| No | 3651 (67.3) | 3535 (96.8) | 3414.1 (93.5) | 3354.2 (91.9) | -120.9 (-3.4) | -180.8 (-5.1) | 0.795 | 0.463 |
|  |  | (95.1—96.6) |  |  |  |  |  |  |
| **ER Negative** |  |  |  |  |  |  |  |  |
| Yes | 103 (1.9) | 57 (55.3) | 92.8 (90.1) | 77.9 (75.6) | 35.8 (62.8) | 20.9 (36.7) | 0.532 | 0.984 |
|  |  | (40.7—61.7) |  |  |  |  |  |  |
| No | 1559 (28.7) | 1471 (94.4) | 1435.9 (92.1) | 1240.2 (79.6) | -35.1 (-2.4) | -230.8 (-15.7) | 0.963 | <0.001 |
|  |  | (91.4—94.3) |  |  |  |  |  |  |
| **Type of surgery** |  |  |  |  |  |  |  |  |
| Mastectomy | 3655 (67.4) | 3448 (94.3) | 3363.4 (92.0) | 3156.7 (86.4) | -84.6 (-2.5) | -291.3 (-8.4) | 0.982 | <0.001 |
|  |  | (92.0—93.9) |  |  |  |  |  |  |
| Breast-conserving | 1756 (32.4) | 1689 (96.2) | 1670.0 (95.1) | 1603.4 (91.3) | -19.0 (-1.1) | -85.6 (-5.1) | 0.988 | 0.594 |
|  |  | (93.6—96.1) |  |  |  |  |  |  |
| **ER Positive** |  |  |  |  |  |  |  |  |
| Mastectomy | 2465 (45.4) | 2357 (95.6) | 2279.6 (92.5) | 2234.3 (90.6) | -77.4 (-3.3) | -122.7 (-5.2) | 0.952 | 0.799 |
|  |  | (93.3—95.4) |  |  |  |  |  |  |
| Breast-conserving | 1287 (23.7) | 1255 (97.5) | 1227.8 (95.4) | 1209.7 (94.0) | -27.2 (-2.2) | -45.3 (-3.6) | 0.982 | 0.963 |
|  |  | (95.4—97.8) |  |  |  |  |  |  |
| **ER Negative** |  |  |  |  |  |  |  |  |
| Mastectomy | 1190 (21.9) | 1091 (91.7) | 1083.8 (91.1) | 922.4 (77.5) | -7.2 (-0.7) | -168.6 (-15.5) | 0.994 | <0.001 |
|  |  | (88.0—91.8) |  |  |  |  |  |  |
| Breast-conserving | 469 (8.6) | 434 (92.5) | 442.2 (94.3) | 393.6 (83.9) | 8.2 (1.9) | -40.4 (-9.3) | 0.996 | 0.639 |
|  |  | (86.6—93.2) |  |  |  |  |  |  |
| **Lymph node procedure** | |  |  |  |  |  |  |  |
| SLNB | 2401 (44.3) | 2338 (97.4) | 2273.3 (94.7) | 2193.4 (91.4) | -64.7 (-2.8) | -144.6 (-6.2) | 0.922 | 0.216 |
|  |  | (95.6—97.3) |  |  |  |  |  |  |
| ALND | 1303 (24.0) | 1188 (91.2) | 1193.3 (91.6) | 1105.9 (84.9) | 5.3 (0.4) | -82.1 (-6.9) | 0.996 | 0.099 |
|  |  | (88.0—91.6) |  |  |  |  |  |  |
| SLNB+ALND | 1464 (27.0) | 1391 (95.0) | 1347.1 (92.0) | 1260.8 (86.1) | -43.9 (-3.2) | -130.2 (-9.4) | 0.920 | 0.027 |
|  |  | (92.0—94.9) |  |  |  |  |  |  |
| Unknown | 256 (4.7) | 232 (90.6) | 231.2 (90.3) | 210.7 (82.3) | -0.8 (-0.3) | -21.3 (-9.2) | 0.993 | 0.533 |
|  |  | (84.7—93.2) |  |  |  |  |  |  |
| **ER Positive** |  |  |  |  |  |  |  |  |
| SLNB | 1740 (32.1) | 1709 (98.2) | 1652.8 (95.0) | 1635.7 (94.0) | -56.2 (-3.3) | -73.3 (-4.3) | 0.881 | 0.894 |
|  |  | (96.6—98.4) |  |  |  |  |  |  |
| ALND | 825 (15.2) | 769 (93.2) | 760.0 (92.1) | 742.4 (90.0) | -9.0 (-1.2) | -26.6 (-3.5) | 1.000 | 0.999 |
|  |  | (90.1—94.1) |  |  |  |  |  |  |
| SLNB+ALND | 1039 (19.2) | 997 (96.0) | 958.9 (92.3) | 933.6 (89.9) | -38.1 (-3.8) | -63.4 (-6.4) | 0.956 | 0.705 |
|  |  | (93.1—96.3) |  |  |  |  |  |  |
| Unknown | 158 (2.9) | 146 (92.4) | 144.4 (91.4) | 140.8 (89.1) | -1.6 (-1.1) | -5.2 (-3.6) | 0.976 | 0.975 |
|  |  | (85.7—95.9) |  |  |  |  |  |  |
| **ER Negative** |  |  |  |  |  |  |  |  |
| SLNB | 661 (12.2) | 629 (95.2) | 620.5 (93.9) | 557.7 (84.4) | -8.5 (-1.4) | -71.3 (-11.3) | 0.983 | 0.283 |
|  |  | (91.4—95.9) |  |  |  |  |  |  |
| ALND | 478 (8.8) | 419 (87.7) | 433.2 (90.6) | 363.4 (76.0) | 14.2 (3.4) | -55.6 (-13.3) | 0.996 | 0.070 |
|  |  | (82.4—89.2) |  |  |  |  |  |  |
| SLNB+ALND | 425 (7.8) | 394 (92.7) | 388.2 (91.3) | 327.2 (77.0) | -5.8 (-1.5) | -66.8 (-17.0) | 0.986 | 0.142 |
|  |  | (87.1—93.7) |  |  |  |  |  |  |
| Unknown | 98 (1.8) | 86 (87.8) | 86.8 (88.6) | 69.9 (71.3) | 0.8 (0.9) | -16.1 (-18.7) | 0.974 | 0.520 |
|  |  | (78.6—93.6) |  |  |  |  |  |  |
| **Hormone Therapy** |  |  |  |  |  |  |  |  |
| Yes | 3574 (65.9) | 3443 (96.3) | 3349.7 (93.7) | 3278.3 (91.7) | -93.3 (-2.7) | -164.7 (-4.8) | 0.928 | 0.582 |
|  |  | (94.5—96.1) |  |  |  |  |  |  |
| No | 1850 (34.1) | 1706 (92.2) | 1695.1 (91.6) | 1492.5 (80.7) | -10.9 (-0.6) | -213.5 (-12.5) | 0.994 | <0.001 |
|  |  | (88.2—91.5) |  |  |  |  |  |  |
| **AI (Aromatase Inhibitors)** | 2415 (44.5) | 2315 (95.9) | 2234.7 (92.5) | 2193.6 (90.8) | -80.3 (-3.5) | -121.4 (-5.2) | 0.906 | 0.728 |
|  |  | (93.6—95.7) |  |  |  |  |  |  |
| **SERM (Selective Estrogen Receptor Modulators)** | 1840 (33.9) | 1782 (96.8) | 1764.6 (95.9) | 1718.4 (93.4) | -17.4 (-1.0) | -63.6 (-3.6) | 0.999 | 0.936 |
|  |  | (95.1—97.1) |  |  |  |  |  |  |
| **Generation chemotherapy** | |  |  |  |  |  |  |  |
| No chemotherapy | 1148 (21.2) | 1084 (94.4) | 1044.6 (91.0) | 1018.9 (88.8) | -39.4 (-3.6) | -65.1 (-6.0) | 0.805 | 0.544 |
|  |  | (90.6—94.3) |  |  |  |  |  |  |
| Generation 2 | 766 (14.1) | 742 (96.9) | 720.9 (94.1) | 695.7 (90.8) | -21.1 (-2.8) | -46.3 (-6.2) | 0.988 | 0.573 |
|  |  | (94.8—97.7) |  |  |  |  |  |  |
| Generation 3 | 3510 (64.7) | 3323 (94.7) | 3279.3 (93.4) | 3056.1 (87.1) | -43.7 (-1.3) | -266.9 (-8.0) | 0.995 | <0.001 |
|  |  | (92.3—94.2) |  |  |  |  |  |  |
| **ER Positive** |  |  |  |  |  |  |  |  |
| No chemotherapy | 990 (18.3) | 954 (96.4) | 908.0 (91.7) | 903.4 (91.3) | -46.0 (-4.8) | -50.6 (-5.3) | 0.804 | 0.924 |
|  |  | (93.6—96.8) |  |  |  |  |  |  |
| Generation 2 | 621 (11.4) | 606 (97.6) | 588.8 (94.8) | 581.1 (93.6) | -17.2 (-2.8) | -24.9 (-4.1) | 0.987 | 0.968 |
|  |  | (95.5—98.5) |  |  |  |  |  |  |
| Generation 3 | 2151 (39.7) | 2061 (95.8) | 2019.3 (93.9) | 1968.1 (91.5) | -41.7 (-2.0) | -92.9 (-4.5) | 0.997 | 0.852 |
|  |  | (93.4—95.7) |  |  |  |  |  |  |
| **ER Negative** |  |  |  |  |  |  |  |  |
| No chemotherapy | 158 (2.9) | 130 (82.3) | 136.6 (86.5) | 115.5 (73.1) | 6.6 (5.1) | -14.5 (-11.2) | 0.937 | 0.492 |
|  |  | (69.4—85.0) |  |  |  |  |  |  |
| Generation 2 | 145 (2.7) | 136 (93.8) | 132.1 (91.1) | 114.6 (79.0) | -3.9 (-2.9) | -21.4 (-15.7) | 0.997 | 0.661 |
|  |  | (88.2—97.4) |  |  |  |  |  |  |
| Generation 3 | 1359 (25.1) | 1262 (92.9) | 1260.0 (92.7) | 1088.0 (80.1) | -2.0 (-0.2) | -174.0 (-13.8) | 0.993 | 0.001 |
|  |  | (89.5—92.9) |  |  |  |  |  |  |
| **Targeted Molecular therapy** | |  |  |  |  |  |  |  |
|  |  |  |  |  |  |  |  |  |
| Yes | 945 (17.4) | 902 (95.4) | 886.1 (93.8) | 805.4 (85.2) | -15.9 (-1.8) | -96.6 (-10.7) | 1.000 | 0.046 |
|  |  | (91.6—95.5) |  |  |  |  |  |  |
| No | 4479 (82.6) | 4247 (94.8) | 4158.7 (92.8) | 3965.4 (88.5) | -88.3 (-2.1) | -281.6 (-6.6) | 0.955 | 0.003 |
|  |  | (92.7—94.3) |  |  |  |  |  |  |
| **ER Positive** |  |  |  |  |  |  |  |  |
| Yes | 460 (8.5) | 447 (97.2) | 435.6 (94.7) | 422.0 (91.7) | -11.4 (-2.6) | -25.0 (-5.6) | 0.995 | 0.942 |
|  |  | (92.9—98.0) |  |  |  |  |  |  |
| No | 3302 (60.9) | 3174 (96.1) | 3080.5 (93.3) | 3030.6 (91.8) | -93.5 (-2.9) | -143.4 (-4.5) | 0.919 | 0.713 |
|  |  | (94.2—95.9) |  |  |  |  |  |  |
| **ER Negative** |  |  |  |  |  |  |  |  |
| Yes | 485 (8.9) | 455 (93.8) | 450.5 (92.9) | 383.3 (79.0) | -4.5 (-1.0) | -71.7 (-15.8) | 1.000 | 0.221 |
|  |  | (88.8—94.7) |  |  |  |  |  |  |
| No | 1177 (21.7) | 1073 (91.2) | 1078.1 (91.6) | 934.8 (79.4) | 5.1 (0.5) | -138.2 (-12.9) | 0.982 | 0.002 |
|  |  | (87.3—91.3) |  |  |  |  |  |  |
| **Radiation therapy** |  |  |  |  |  |  |  |  |
| Yes | 1965 (36.2) | 1840 (93.6) | 1847.2 (94.0) | 1715.1 (87.3) | 7.2 (0.4) | -124.9 (-6.8) | 0.974 | 0.053 |
|  |  | (90.8—93.5) |  |  |  |  |  |  |
| No | 3459 (63.8) | 3309 (95.7) | 3197.6 (92.4) | 3055.6 (88.3) | -111.4 (-3.4) | -253.4 (-7.7) | 0.790 | 0.002 |
|  |  | (93.5—95.3) |  |  |  |  |  |  |
| **ER Positive** |  |  |  |  |  |  |  |  |
| Yes | 1358 (25.0) | 1298 (95.6) | 1284.7 (94.6) | 1246.8 (91.8) | -13.3 (-1.0) | -51.2 (-3.9) | 0.996 | 0.919 |
|  |  | (92.8—95.7) |  |  |  |  |  |  |
| No | 2404 (44.3) | 2323 (96.6) | 2231.5 (92.8) | 2205.8 (91.8) | -91.5 (-3.9) | -117.2 (-5.0) | 0.871 | 0.736 |
|  |  | (94.6—96.6) |  |  |  |  |  |  |
| **ER Negative** |  |  |  |  |  |  |  |  |
| Yes | 607 (11.2) | 542 (89.3) | 562.6 (92.7) | 468.3 (77.1) | 20.6 (3.8) | -73.7 (-13.6) | 0.893 | 0.104 |
|  |  | (84.5—90.3) |  |  |  |  |  |  |
| No | 1055 (19.5) | 986 (93.5) | 966.1 (91.6) | 849.8 (80.5) | -19.9 (-2.0) | -136.2 (-13.8) | 0.966 | 0.003 |
|  |  | (89.7—93.6) |  |  |  |  |  |  |

Abbreviations: N = total number, CI = confidence interval, ER = oestrogen receptor, PR = progesterone receptor, HR = Hormone Receptor, HER2 = human epidermal growth factor receptor 2.

*The p-value was calculated by using a Chi squared-test. p-values indicated in bold are considered as statistically significant (p < 0.05). >5% difference was considered as clinically relevant.
