# Supplementary material for: Insights into the performance of PREDICT tool in a large Mainland Chinese breast cancer cohort: a comparative analysis of versions 3.0 and 2.2
Source: Oncologist. 2024 Jun 29;29(8):e976–83. doi: 10.1093/oncolo/oyae164 (PMC11299932; doi:10.1093/oncolo/oyae164)
Supplement: oyae164_suppl_Supplementary_Table_S2 [file oyae164_suppl_supplementary_table_s2.docx]

**Supplementary Table 2**

| **Table S2. Baseline characteristic and observed and expected 10-year survival.** | | | | | | | | |
| --- | --- | --- | --- | --- | --- | --- | --- | --- |
|  | **Numbers and Observed (%)** | | **PREDICT Predict (%)** | | **Difference (%)** | | **P-value** | |
|  | **n (%)** | **Observed** | **v3.0** | **v2.2** | **v3.0** | **v2.2** | **v3.0** | **v2.2** |
|  |  | **(%, 95% CI)** |  |  |  |  |  |  |
| **All Patients** | 517 (100.0) | 457 (88.4) | 446.0 (86.3) | 406.1 (78.5) | -11.0 (-2.4) | -50.9 (-11.1) | 0.860 | 0.023 |
|  |  | (83.2—89.6) |  |  |  |  |  |  |
| **ER Positive** | 340 (65.8) | 300 (88.2) | 295.6 (86.9) | 277.2 (81.5) | -4.4 (-1.5) | -22.8 (-7.6) | 0.982 | 0.855 |
|  |  | (82.3—90.2) |  |  |  |  |  |  |
| **ER Negative** | 177 (34.2) | 157 (88.7) | 150.4 (85.0) | 128.8 (72.8) | -6.6 (-4.2) | -28.2 (-18.0) | 0.522 | 0.002 |
|  |  | (81.3—92.3) |  |  |  |  |  |  |
| **PR status** |  |  |  |  |  |  |  |  |
| Positive | 289 (55.9) | 257 (88.9) | 253.9 (87.9) | 236.7 (81.9) | -3.1 (-1.2) | -20.3 (-7.9) | 0.964 | 0.930 |
|  |  | (82.9—91.3) |  |  |  |  |  |  |
| Negative | 225 (43.5) | 197 (87.6) | 189.4 (84.2) | 166.9 (74.2) | -7.6 (-3.9) | -30.1 (-15.3) | 0.614 | 0.004 |
|  |  | (80.4—90.5) |  |  |  |  |  |  |
| Unknown | 3 (0.6) | 3 (100.0) | 2.7 (90.0) | 2.5 (83.3) | -0.3 (-10.0) | -0.5 (-16.7) | 0.999 | 0.998 |
|  |  | (100.0—100.0) |  |  |  |  |  |  |
| **ER Positive** |  |  |  |  |  |  |  |  |
| Positive | 275 (53.2) | 247 (89.8) | 241.5 (87.8) | 226.7 (82.4) | -5.5 (-2.2) | -20.3 (-8.2) | 0.981 | 0.962 |
|  |  | (83.9—92.2) |  |  |  |  |  |  |
| Negative | 63 (12.2) | 51 (81.0) | 52.3 (83.0) | 48.9 (77.6) | 1.3 (2.5) | -2.1 (-4.1) | 0.995 | 0.821 |
|  |  | (67.8—89.9) |  |  |  |  |  |  |
| Unknown | 2 (0.4) | 2 (100.0) | 1.8 (90.0) | 1.7 (85.0) | -0.2 (-10.0) | -0.3 (-15.0) | 0.983 | 0.963 |
|  |  | (100.0—100.0) |  |  |  |  |  |  |
| **ER Negative** |  |  |  |  |  |  |  |  |
| Positive | 14 (2.7) | 10 (71.4) | 12.4 (88.6) | 10.0 (71.4) | 2.4 (24.0) | 0.0 (0.0) | 0.839 | 0.743 |
|  |  | (51.3—99.5) |  |  |  |  |  |  |
| Negative | 162 (31.3) | 146 (90.1) | 137.1 (84.6) | 118.0 (72.8) | -8.9 (-6.1) | -28.0 (-19.2) | 0.581 | 0.002 |
|  |  | (82.9—93.8) |  |  |  |  |  |  |
| Unknown | 1 (0.2) | 1 (100.0) | 0.9 (90.0) | 0.8 (80.0) | -0.1 (-10.0) | -0.2 (-20.0) | NA | NA |
|  |  |  |  |  |  |  |  |  |
| **HER2 status** |  |  |  |  |  |  |  |  |
| Positive | 138 (26.7) | 125 (90.6) | 116.8 (84.6) | 99.3 (72.0) | -8.2 (-6.6) | -25.7 (-20.6) | 0.739 | 0.024 |
|  |  | (82.8—94.6) |  |  |  |  |  |  |
| Negative | 297 (57.4) | 261 (87.9) | 258.6 (87.1) | 241.7 (81.4) | -2.4 (-0.9) | -19.3 (-7.4) | 0.942 | 0.607 |
|  |  | (81.6—90.2) |  |  |  |  |  |  |
| Low | 82 (15.9) | 71 (86.6) | 70.6 (86.1) | 65.1 (79.4) | -0.4 (-0.6) | -5.9 (-8.3) | 0.950 | 0.945 |
|  |  | (76.9—93.5) |  |  |  |  |  |  |
| **ER Positive** |  |  |  |  |  |  |  |  |
| Positive | 60 (11.6) | 54 (90.0) | 51.6 (86.0) | 45.7 (76.2) | -2.4 (-4.4) | -8.3 (-15.4) | 0.864 | 0.765 |
|  |  | (79.5—97.5) |  |  |  |  |  |  |
| Negative | 217 (42.0) | 191 (88.0) | 189.7 (87.4) | 179.9 (82.9) | -1.3 (-0.7) | -11.1 (-5.8) | 0.960 | 0.994 |
|  |  | (81.1—91.1) |  |  |  |  |  |  |
| Low | 63 (12.2) | 55 (87.3) | 54.4 (86.3) | 51.6 (81.9) | -0.6 (-1.1) | -3.4 (-6.2) | 0.907 | 0.999 |
|  |  | (75.8—95.2) |  |  |  |  |  |  |
| **ER Negative** |  |  |  |  |  |  |  |  |
| Positive | 78 (15.1) | 71 (91.0) | 65.2 (83.6) | 53.6 (68.7) | -5.8 (-8.2) | -17.4 (-24.5) | 0.899 | 0.076 |
|  |  | (81.4—97.0) |  |  |  |  |  |  |
| Negative | 80 (15.5) | 70 (87.5) | 69.0 (86.2) | 61.7 (77.1) | -1.0 (-1.4) | -8.3 (-11.9) | 0.808 | 0.234 |
|  |  | (77.1—94.1) |  |  |  |  |  |  |
| Low | 19 (3.7) | 16 (84.2) | 16.3 (85.8) | 13.5 (71.1) | 0.3 (1.9) | -2.5 (-15.6) | 0.817 | 0.654 |
|  |  | (67.8—100.0) |  |  |  |  |  |  |
| **Grade** |  |  |  |  |  |  |  |  |
| 1 | 34 (6.6) | 30 (88.2) | 30.6 (90.0) | 30.1 (88.5) | 0.6 (2.0) | 0.1 (0.3) | 0.998 | 0.998 |
|  |  | (74.6—99.7) |  |  |  |  |  |  |
| 2 | 181 (35.0) | 166 (91.7) | 154.9 (85.6) | 136.8 (75.6) | -11.1 (-6.7) | -29.2 (-17.6) | 0.931 | 0.042 |
|  |  | (85.4—95.0) |  |  |  |  |  |  |
| 3 | 84 (16.2) | 74 (88.1) | 71.2 (84.8) | 60.4 (71.9) | -2.8 (-3.8) | -13.6 (-18.4) | 0.936 | 0.731 |
|  |  | (79.1—94.7) |  |  |  |  |  |  |
| Unknown | 218 (42.2) | 187 (85.8) | 189.4 (86.9) | 178.8 (82.0) | 2.4 (1.3) | -8.2 (-4.4) | 0.865 | 0.824 |
|  |  | (78.0—88.8) |  |  |  |  |  |  |
| **ER Positive** |  |  |  |  |  |  |  |  |
| 1 | 28 (5.4) | 25 (89.3) | 25.0 (89.3) | 24.8 (88.6) | 0.0 (0.0) | -0.2 (-0.8) | 0.999 | 0.998 |
|  |  | (76.7—100.0) |  |  |  |  |  |  |
| 2 | 122 (23.6) | 110 (90.2) | 106.5 (87.3) | 100.4 (82.3) | -3.5 (-3.2) | -9.6 (-8.7) | 0.985 | 0.983 |
|  |  | (82.3—94.8) |  |  |  |  |  |  |
| 3 | 39 (7.5) | 34 (87.2) | 32.7 (83.8) | 29.4 (75.4) | -1.3 (-3.8) | -4.6 (-13.5) | 0.994 | 0.978 |
|  |  | (73.4—98.0) |  |  |  |  |  |  |
| Unknown | 151 (29.2) | 131 (86.8) | 131.4 (87.0) | 122.6 (81.2) | 0.4 (0.3) | -8.4 (-6.4) | 0.974 | 0.847 |
|  |  | (78.3—90.9) |  |  |  |  |  |  |
| **ER Negative** |  |  |  |  |  |  |  |  |
| 1 | 6 (1.2) | 5 (83.3) | 5.6 (93.3) | 5.3 (88.3) | 0.6 (12.0) | 0.3 (6.0) | 0.901 | 0.888 |
|  |  | (42.6—100.0) |  |  |  |  |  |  |
| 2 | 59 (11.4) | 56 (94.9) | 48.4 (82.0) | 36.3 (61.5) | -7.6 (-13.6) | -19.7 (-35.2) | 0.490 | 0.050 |
|  |  | (87.0—100.0) |  |  |  |  |  |  |
| 3 | 45 (8.7) | 40 (88.9) | 38.5 (85.6) | 31.0 (68.9) | -1.5 (-3.8) | -9.0 (-22.5) | 0.946 | 0.509 |
|  |  | (78.8—98.5) |  |  |  |  |  |  |
| Unknown | 67 (13.0) | 56 (83.6) | 57.9 (86.4) | 56.2 (83.9) | 1.9 (3.4) | 0.2 (0.4) | 0.974 | 0.966 |
|  |  | (70.8—91.6) |  |  |  |  |  |  |
| 70-85 | 32 (6.2) | 20 (62.5) | 17.2 (53.8) | 16.6 (51.9) | -2.8 (-14.0) | -3.4 (-17.0) | 0.032 | <0.001 |
|  |  | (36.0—76.1) |  |  |  |  |  |  |
| **ER Positive** |  |  |  |  |  |  |  |  |
| <40 | 40 (7.7) | 31 (77.5) | 36.6 (91.5) | 33.0 (82.5) | 5.6 (18.1) | 2.0 (6.5) | 0.966 | 0.908 |
|  |  | (60.2—90.1) |  |  |  |  |  |  |
| 40-49 | 138 (26.7) | 129 (93.5) | 126.3 (91.5) | 117.5 (85.1) | -2.7 (-2.1) | -11.5 (-8.9) | 0.999 | 0.986 |
|  |  | (87.5—97.2) |  |  |  |  |  |  |
| 50-59 | 92 (17.8) | 86 (93.5) | 81.3 (88.4) | 76.6 (83.3) | -4.7 (-5.5) | -9.4 (-10.9) | 1.000 | 0.987 |
|  |  | (87.0—98.5) |  |  |  |  |  |  |
| 60-69 | 49 (9.5) | 43 (87.8) | 39.2 (80.0) | 37.5 (76.5) | -3.8 (-8.8) | -5.5 (-12.8) | 0.973 | 0.997 |
|  |  | (74.9—96.9) |  |  |  |  |  |  |
| 70-85 | 21 (4.1) | 11 (52.4) | 12.2 (58.1) | 12.7 (60.5) | 1.2 (10.9) | 1.7 (15.5) | 0.323 | 0.169 |
|  |  | (26.8—74.9) |  |  |  |  |  |  |
| **ER Negative** |  |  |  |  |  |  |  |  |
| <40 | 21 (4.1) | 18 (85.7) | 18.9 (90.0) | 16.6 (79.0) | 0.9 (5.0) | -1.4 (-7.8) | 0.989 | 0.831 |
|  |  | (67.8—100.0) |  |  |  |  |  |  |
| 40-49 | 57 (11.0) | 50 (87.7) | 51.4 (90.2) | 44.8 (78.6) | 1.4 (2.8) | -5.2 (-10.4) | 0.996 | 0.673 |
|  |  | (75.4—95.9) |  |  |  |  |  |  |
| 50-59 | 62 (12.0) | 55 (88.7) | 54.5 (87.9) | 45.6 (73.5) | -0.5 (-0.9) | -9.4 (-17.1) | 0.978 | 0.394 |
|  |  | (79.6—96.7) |  |  |  |  |  |  |
| 60-69 | 26 (5.0) | 25 (96.2) | 20.6 (79.2) | 17.8 (68.5) | -4.4 (-17.6) | -7.2 (-28.8) | 1.000 | 0.875 |
|  |  | (86.6—100.0) |  |  |  |  |  |  |
| 70-85 | 11 (2.1) | 9 (81.8) | 5.0 (45.5) | 4.0 (36.4) | -4.0 (-44.4) | -5.0 (-55.6) | 0.320 | 0.010 |
|  |  | (44.7—100.0) |  |  |  |  |  |  |
| **Tumor size(mm)** | |  |  |  |  |  |  |  |
| 0-9 | 20 (3.9) | 15 (75.0) | 18.1 (90.5) | 17.7 (88.5) | 3.1 (20.7) | 2.7 (18.0) | 0.988 | 0.884 |
|  |  | (53.1—96.2) |  |  |  |  |  |  |
| 10-19 | 141 (27.3) | 131 (92.9) | 124.9 (88.6) | 116.8 (82.8) | -6.1 (-4.7) | -14.2 (-10.8) | 0.965 | 0.856 |
|  |  | (87.8—97.0) |  |  |  |  |  |  |
| 20-29 | 170 (32.9) | 153 (90.0) | 149.4 (87.9) | 137.1 (80.6) | -3.6 (-2.4) | -15.9 (-10.4) | 0.950 | 0.760 |
|  |  | (82.8—93.5) |  |  |  |  |  |  |
| 30-49 | 102 (19.7) | 90 (88.2) | 84.7 (83.0) | 73.4 (72.0) | -5.3 (-5.9) | -16.6 (-18.4) | 0.962 | 0.265 |
|  |  | (78.2—93.4) |  |  |  |  |  |  |
| 50+ | 18 (3.5) | 15 (83.3) | 13.3 (73.9) | 10.7 (59.4) | -1.7 (-11.3) | -4.3 (-28.7) | 0.646 | 0.348 |
|  |  | (60.0—100.0) |  |  |  |  |  |  |
| Unknown | 66 (12.8) | 53 (80.3) | 55.6 (84.2) | 50.4 (76.4) | 2.6 (4.9) | -2.6 (-4.9) | 0.999 | 0.373 |
|  |  | (65.0—88.2) |  |  |  |  |  |  |
| **ER Positive** |  |  |  |  |  |  |  |  |
| 0-9 | 13 (2.5) | 10 (76.9) | 11.8 (90.8) | 11.6 (89.2) | 1.8 (18.0) | 1.6 (16.0) | 0.974 | 0.972 |
|  |  | (57.1—100.0) |  |  |  |  |  |  |
| 10-19 | 91 (17.6) | 86 (94.5) | 81.6 (89.7) | 79.3 (87.1) | -4.4 (-5.1) | -6.7 (-7.8) | 0.995 | 0.992 |
|  |  | (89.2—99.3) |  |  |  |  |  |  |
| 20-29 | 119 (23.0) | 109 (91.6) | 104.5 (87.8) | 97.6 (82.0) | -4.5 (-4.1) | -11.4 (-10.5) | 0.982 | 0.995 |
|  |  | (83.5—95.9) |  |  |  |  |  |  |
| 30-49 | 62 (12.0) | 53 (85.5) | 51.2 (82.6) | 45.8 (73.9) | -1.8 (-3.4) | -7.2 (-13.6) | 0.963 | 0.781 |
|  |  | (72.8—93.6) |  |  |  |  |  |  |
| 50+ | 12 (2.3) | 9 (75.0) | 8.5 (70.8) | 7.0 (58.3) | -0.5 (-5.6) | -2.0 (-22.2) | 0.509 | 0.218 |
|  |  | (39.3—100.0) |  |  |  |  |  |  |
| Unknown | 43 (8.3) | 33 (76.7) | 38.2 (88.8) | 36.0 (83.7) | 5.2 (15.8) | 3.0 (9.1) | 0.674 | 0.825 |
|  |  | (59.1—88.6) |  |  |  |  |  |  |
| **ER Negative** |  |  |  |  |  |  |  |  |
| 0-9 | 7 (1.4) | 5 (71.4) | 6.3 (90.0) | 6.1 (87.1) | 1.3 (26.0) | 1.1 (22.0) | 0.979 | 0.879 |
|  |  | (21.4—100.0) |  |  |  |  |  |  |
| 10-19 | 50 (9.7) | 45 (90.0) | 43.3 (86.6) | 37.5 (75.0) | -1.7 (-3.8) | -7.5 (-16.7) | 0.947 | 0.623 |
|  |  | (80.2—98.6) |  |  |  |  |  |  |
| 20-29 | 51 (9.9) | 44 (86.3) | 45.0 (88.2) | 39.5 (77.5) | 1.0 (2.3) | -4.5 (-10.2) | 0.876 | 0.630 |
|  |  | (75.1—95.9) |  |  |  |  |  |  |
| 30-49 | 40 (7.7) | 37 (92.5) | 33.6 (84.0) | 27.6 (69.0) | -3.4 (-9.2) | -9.4 (-25.4) | 0.997 | 0.436 |
|  |  | (80.7—100.0) |  |  |  |  |  |  |
| 50+ | 6 (1.2) | 6 (100.0) | 4.8 (80.0) | 3.7 (61.7) | -1.2 (-20.0) | -2.3 (-38.3) | 1.000 | 0.988 |
|  |  | (100.0—100.0) |  |  |  |  |  |  |
| Unknown | 23 (4.4) | 20 (87.0) | 17.4 (75.7) | 14.4 (62.6) | -2.6 (-13.0) | -5.6 (-28.0) | 0.307 | 0.040 |
|  |  | (66.6—100.0) |  |  |  |  |  |  |
| **Nodes positive** |  |  |  |  |  |  |  |  |
| 0 | 307 (59.4) | 274 (89.3) | 272.4 (88.7) | 260.5 (84.9) | -1.6 (-0.6) | -13.5 (-4.9) | 0.983 | 0.962 |
|  |  | (83.9—91.7) |  |  |  |  |  |  |
| 1 | 51 (9.9) | 46 (90.2) | 45.1 (88.4) | 41.9 (82.2) | -0.9 (-2.0) | -4.1 (-8.9) | 0.990 | 0.971 |
|  |  | (78.0—98.4) |  |  |  |  |  |  |
| 2-4 | 76 (14.7) | 66 (86.8) | 65.3 (85.9) | 57.3 (75.4) | -0.7 (-1.1) | -8.7 (-13.2) | 0.889 | 0.832 |
|  |  | (75.1—93.5) |  |  |  |  |  |  |
| 5-9 | 50 (9.7) | 41 (82.0) | 40.3 (80.6) | 30.8 (61.6) | -0.7 (-1.7) | -10.2 (-24.9) | 0.952 | 0.264 |
|  |  | (70.0—92.8) |  |  |  |  |  |  |
| 10+ | 33 (6.4) | 30 (90.9) | 22.9 (69.4) | 15.6 (47.3) | -7.1 (-23.7) | -14.4 (-48.0) | 0.389 | 0.004 |
|  |  | (71.3—100.0) |  |  |  |  |  |  |
| **ER Positive** |  |  |  |  |  |  |  |  |
| 0 | 204 (39.5) | 185 (90.7) | 181.7 (89.1) | 177.0 (86.8) | -3.3 (-1.8) | -8.0 (-4.3) | 0.991 | 0.925 |
|  |  | (85.1—94.1) |  |  |  |  |  |  |
| 1 | 34 (6.6) | 32 (94.1) | 30.4 (89.4) | 28.7 (84.4) | -1.6 (-5.0) | -3.3 (-10.3) | 0.983 | 0.982 |
|  |  | (83.2—100.0) |  |  |  |  |  |  |
| 2-4 | 50 (9.7) | 40 (80.0) | 42.8 (85.6) | 39.4 (78.8) | 2.8 (7.0) | -0.6 (-1.5) | 0.962 | 0.766 |
|  |  | (61.6—89.4) |  |  |  |  |  |  |
| 5-9 | 34 (6.6) | 27 (79.4) | 27.4 (80.6) | 22.2 (65.3) | 0.4 (1.5) | -4.8 (-17.8) | 0.986 | 0.907 |
|  |  | (64.2—93.7) |  |  |  |  |  |  |
| 10+ | 18 (3.5) | 16 (88.9) | 13.3 (73.9) | 10.0 (55.6) | -2.7 (-16.9) | -6.0 (-37.5) | 0.879 | 0.654 |
|  |  | (67.1—100.0) |  |  |  |  |  |  |
| **ER Negative** |  |  |  |  |  |  |  |  |
| 0 | 103 (19.9) | 89 (86.4) | 90.8 (88.2) | 83.5 (81.1) | 1.8 (2.0) | -5.5 (-6.2) | 0.768 | 0.794 |
|  |  | (77.0—92.2) |  |  |  |  |  |  |
| 1 | 17 (3.3) | 14 (82.4) | 14.7 (86.5) | 13.2 (77.6) | 0.7 (5.0) | -0.8 (-5.7) | 0.944 | 0.867 |
|  |  | (57.1—100.0) |  |  |  |  |  |  |
| 2-4 | 26 (5.0) | 26 (100.0) | 22.5 (86.5) | 17.9 (68.8) | -3.5 (-13.5) | -8.1 (-31.2) | 1.000 | 0.939 |
|  |  | (100.0—100.0) |  |  |  |  |  |  |
| 5-9 | 16 (3.1) | 14 (87.5) | 12.8 (80.0) | 8.5 (53.1) | -1.2 (-8.6) | -5.5 (-39.3) | 0.983 | 0.870 |
|  |  | (71.1—100.0) |  |  |  |  |  |  |
| 10+ | 15 (2.9) | 14 (93.3) | 9.5 (63.3) | 5.6 (37.3) | -4.5 (-32.1) | -8.4 (-60.0) | 0.456 | 0.016 |
|  |  | (67.3—100.0) |  |  |  |  |  |  |
| **Menopasus status** | |  |  |  |  |  |  |  |
| Pre | 95 (18.4) | 83 (87.4) | 86.8 (91.4) | 78.9 (83.1) | 3.8 (4.6) | -4.1 (-4.9) | 0.943 | 0.810 |
|  |  | (78.7—93.6) |  |  |  |  |  |  |
| Post | 149 (28.8) | 129 (86.6) | 115.0 (77.2) | 107.0 (71.8) | -14.0 (-10.9) | -22.0 (-17.1) | 0.928 | 0.072 |
|  |  | (78.1—90.8) |  |  |  |  |  |  |
| Unknown | 273 (52.8) | 245 (89.7) | 244.2 (89.5) | 220.1 (80.6) | -0.8 (-0.3) | -24.9 (-10.2) | 0.968 | 0.132 |
|  |  | (83.6—92.1) |  |  |  |  |  |  |
| **ER Positive** |  |  |  |  |  |  |  |  |
| Pre | 66 (12.8) | 57 (86.4) | 60.6 (91.8) | 55.6 (84.2) | 3.6 (6.3) | -1.4 (-2.5) | 0.997 | 0.886 |
|  |  | (76.2—94.5) |  |  |  |  |  |  |
| Post | 102 (19.7) | 85 (83.3) | 80.4 (78.8) | 77.6 (76.1) | -4.6 (-5.4) | -7.4 (-8.7) | 0.947 | 0.992 |
|  |  | (73.3—89.6) |  |  |  |  |  |  |
| Unknown | 172 (33.3) | 158 (91.9) | 154.7 (89.9) | 144.1 (83.8) | -3.3 (-2.1) | -13.9 (-8.8) | 0.996 | 0.934 |
|  |  | (85.3—95.2) |  |  |  |  |  |  |
| **ER Negative** |  |  |  |  |  |  |  |  |
| Pre | 29 (5.6) | 26 (89.7) | 26.3 (90.7) | 23.3 (80.3) | 0.3 (1.2) | -2.7 (-10.4) | 0.970 | 0.852 |
|  |  | (76.7—100.0) |  |  |  |  |  |  |
| Post | 47 (9.1) | 44 (93.6) | 34.6 (73.6) | 29.5 (62.8) | -9.4 (-21.4) | -14.5 (-33.0) | 0.556 | 0.006 |
|  |  | (83.6—100.0) |  |  |  |  |  |  |
| Unknown | 101 (19.5) | 87 (86.1) | 89.6 (88.7) | 76.0 (75.2) | 2.6 (3.0) | -11.0 (-12.6) | 0.919 | 0.222 |
|  |  | (76.5—92.0) |  |  |  |  |  |  |
| **Pathologic T** |  |  |  |  |  |  |  |  |
| T1 | 277 (53.6) | 252 (91.0) | 245.0 (88.4) | 228.7 (82.6) | -7.0 (-2.8) | -23.3 (-9.2) | 0.933 | 0.550 |
|  |  | (85.9—93.6) |  |  |  |  |  |  |
| T2 | 165 (31.9) | 146 (88.5) | 138.1 (83.7) | 121.1 (73.4) | -7.9 (-5.4) | -24.9 (-17.1) | 0.749 | 0.037 |
|  |  | (80.4—92.1) |  |  |  |  |  |  |
| T3 | 8 (1.5) | 5 (62.5) | 6.4 (80.0) | 5.3 (66.2) | 1.4 (28.0) | 0.3 (6.0) | 0.751 | 0.709 |
|  |  | (30.1—100.0) |  |  |  |  |  |  |
| T4 | 4 (0.8) | 3 (75.0) | 2.6 (65.0) | 1.9 (47.5) | -0.4 (-13.3) | -1.1 (-36.7) | 0.080 | <0.001 |
|  |  | / |  |  |  |  |  |  |
| Unknown | 63 (12.2) | 51 (81.0) | 53.9 (85.6) | 49.0 (77.8) | 2.9 (5.7) | -2.0 (-3.9) | 0.912 | 0.885 |
|  |  | (66.6—89.4) |  |  |  |  |  |  |
| **ER Positive** |  |  |  |  |  |  |  |  |
| T1 | 185 (35.8) | 171 (92.4) | 164.3 (88.8) | 157.1 (84.9) | -6.7 (-3.9) | -13.9 (-8.1) | 0.990 | 0.980 |
|  |  | (87.1—95.8) |  |  |  |  |  |  |
| T2 | 108 (20.9) | 95 (88.0) | 90.0 (83.3) | 81.4 (75.4) | -5.0 (-5.3) | -13.6 (-14.3) | 0.948 | 0.532 |
|  |  | (78.1—93.0) |  |  |  |  |  |  |
| T3 | 4 (0.8) | 1 (25.0) | 3.1 (77.5) | 2.7 (67.5) | 2.1 (210.0) | 1.7 (170.0) | 0.461 | 0.402 |
|  |  | / |  |  |  |  |  |  |
| T4 | 1 (0.2) | 1 (100.0) | 0.7 (70.0) | 0.6 (60.0) | -0.3 (-30.0) | -0.4 (-40.0) | NA | NA |
|  |  | / |  |  |  |  |  |  |
| Unknown | 42 (8.1) | 32 (76.2) | 37.5 (89.3) | 35.4 (84.3) | 5.5 (17.2) | 3.4 (10.6) | 0.665 | 0.573 |
|  |  | (59.1—88.6) |  |  |  |  |  |  |
| **ER Negative** |  |  |  |  |  |  |  |  |
| T1 | 92 (17.8) | 81 (88.0) | 80.7 (87.7) | 71.6 (77.8) | -0.3 (-0.4) | -9.4 (-11.6) | 0.857 | 0.335 |
|  |  | (79.0—94.1) |  |  |  |  |  |  |
| T2 | 57 (11.0) | 51 (89.5) | 48.1 (84.4) | 39.7 (69.6) | -2.9 (-5.7) | -11.3 (-22.2) | 0.856 | 0.138 |
|  |  | (78.8—97.4) |  |  |  |  |  |  |
| T3 | 4 (0.8) | 4 (100.0) | 3.3 (82.5) | 2.6 (65.0) | -0.7 (-17.5) | -1.4 (-35.0) | 1.000 | 0.960 |
|  |  | (100.0—100.0) |  |  |  |  |  |  |
| T4 | 3 (0.6) | 2 (66.7) | 1.9 (63.3) | 1.4 (46.7) | -0.1 (-5.0) | -0.6 (-30.0) | 0.026 | <0.001 |
|  |  | / |  |  |  |  |  |  |
| Unknown | 21 (4.1) | 19 (90.5) | 16.4 (78.1) | 13.6 (64.8) | -2.6 (-13.7) | -5.4 (-28.4) | 0.700 | 0.303 |
|  |  | (73.4—100.0) |  |  |  |  |  |  |
| **Pathologic N** |  |  |  |  |  |  |  |  |
| N0 | 307 (59.4) | 274 (89.3) | 272.4 (88.7) | 260.4 (84.8) | -1.6 (-0.6) | -13.6 (-5.0) | 0.983 | 0.961 |
|  |  | (83.8—91.7) |  |  |  |  |  |  |
| N1mi | 2 (0.4) | 2 (100.0) | 1.8 (90.0) | 1.6 (80.0) | -0.2 (-10.0) | -0.4 (-20.0) | 0.936 | 0.911 |
|  |  | (100.0—100.0) |  |  |  |  |  |  |
| N1 | 106 (20.5) | 94 (88.7) | 92.2 (87.0) | 83.0 (78.3) | -1.8 (-1.9) | -11.0 (-11.7) | 0.922 | 0.772 |
|  |  | (79.2—93.8) |  |  |  |  |  |  |
| N2 | 67 (13.0) | 55 (82.1) | 55.2 (82.4) | 43.8 (65.4) | 0.2 (0.4) | -11.2 (-20.4) | 0.978 | 0.445 |
|  |  | (70.1—90.7) |  |  |  |  |  |  |
| N3 | 35 (6.8) | 32 (91.4) | 24.4 (69.7) | 17.2 (49.1) | -7.6 (-23.8) | -14.8 (-46.2) | 0.371 | 0.002 |
|  |  | (73.6—100.0) |  |  |  |  |  |  |
| **ER Positive** |  |  |  |  |  |  |  |  |
| N0 | 204 (39.5) | 185 (90.7) | 181.6 (89.0) | 176.9 (86.7) | -3.4 (-1.8) | -8.1 (-4.4) | 0.991 | 0.924 |
|  |  | (85.0—94.0) |  |  |  |  |  |  |
| N1mi | 2 (0.4) | 2 (100.0) | 1.8 (90.0) | 1.6 (80.0) | -0.2 (-10.0) | -0.4 (-20.0) | 0.936 | 0.911 |
|  |  | (100.0—100.0) |  |  |  |  |  |  |
| N1 | 69 (13.3) | 60 (87.0) | 60.1 (87.1) | 56.2 (81.4) | 0.1 (0.2) | -3.8 (-6.3) | 0.976 | 0.964 |
|  |  | (74.1—94.0) |  |  |  |  |  |  |
| N2 | 45 (8.7) | 35 (77.8) | 37.0 (82.2) | 30.8 (68.4) | 2.0 (5.7) | -4.2 (-12.0) | 0.979 | 0.824 |
|  |  | (62.2—89.5) |  |  |  |  |  |  |
| N3 | 20 (3.9) | 18 (90.0) | 15.2 (76.0) | 11.7 (58.5) | -2.8 (-15.6) | -6.3 (-35.0) | 0.887 | 0.588 |
|  |  | (71.1—100.0) |  |  |  |  |  |  |
| **ER Negative** |  |  |  |  |  |  |  |  |
| N0 | 103 (19.9) | 89 (86.4) | 90.8 (88.2) | 83.5 (81.1) | 1.8 (2.0) | -5.5 (-6.2) | 0.768 | 0.794 |
|  |  | (77.0—92.2) |  |  |  |  |  |  |
| N1 | 37 (7.2) | 34 (91.9) | 32.1 (86.8) | 26.8 (72.4) | -1.9 (-5.6) | -7.2 (-21.2) | 0.998 | 0.719 |
|  |  | (81.1—100.0) |  |  |  |  |  |  |
| N2 | 22 (4.3) | 20 (90.9) | 18.3 (83.2) | 13.1 (59.5) | -1.7 (-8.5) | -6.9 (-34.5) | 0.990 | 0.847 |
|  |  | (77.8—100.0) |  |  |  |  |  |  |
| N3 | 15 (2.9) | 14 (93.3) | 9.2 (61.3) | 5.5 (36.7) | -4.8 (-34.3) | -8.5 (-60.7) | 0.478 | 0.016 |
|  |  | (67.3—100.0) |  |  |  |  |  |  |
| **AJCC stage** |  |  |  |  |  |  |  |  |
| I | 186 (36.0) | 170 (91.4) | 167.1 (89.8) | 160.6 (86.3) | -2.9 (-1.7) | -9.4 (-5.5) | 0.994 | 0.942 |
|  |  | (86.1—95.0) |  |  |  |  |  |  |
| II | 178 (34.4) | 155 (87.1) | 154.3 (86.7) | 141.8 (79.7) | -0.7 (-0.5) | -13.2 (-8.5) | 0.938 | 0.885 |
|  |  | (78.7—90.5) |  |  |  |  |  |  |
| III | 104 (20.1) | 89 (85.6) | 81.4 (78.3) | 62.7 (60.3) | -7.6 (-8.5) | -26.3 (-29.6) | 0.279 | <0.001 |
|  |  | (73.9—90.6) |  |  |  |  |  |  |
| Unknown | 49 (9.5) | 43 (87.8) | 43.2 (88.2) | 41.0 (83.7) | 0.2 (0.5) | -2.0 (-4.7) | 0.982 | 0.981 |
|  |  | (75.5—97.0) |  |  |  |  |  |  |
| **ER Positive** |  |  |  |  |  |  |  |  |
| I | 124 (24.0) | 118 (95.2) | 111.9 (90.2) | 109.5 (88.3) | -6.1 (-5.2) | -8.5 (-7.2) | 0.999 | 0.996 |
|  |  | (90.4—98.9) |  |  |  |  |  |  |
| II | 116 (22.4) | 98 (84.5) | 99.8 (86.0) | 94.7 (81.6) | 1.8 (1.8) | -3.3 (-3.4) | 0.973 | 0.995 |
|  |  | (73.6—89.4) |  |  |  |  |  |  |
| III | 65 (12.6) | 53 (81.5) | 52.1 (80.2) | 42.5 (65.4) | -0.9 (-1.7) | -10.5 (-19.8) | 0.694 | 0.186 |
|  |  | (67.0—89.6) |  |  |  |  |  |  |
| Unknown | 35 (6.8) | 31 (88.6) | 31.7 (90.6) | 30.6 (87.4) | 0.7 (2.3) | -0.4 (-1.3) | 0.962 | 0.992 |
|  |  | (74.6—99.8) |  |  |  |  |  |  |
| **ER Negative** |  |  |  |  |  |  |  |  |
| I | 62 (12.0) | 52 (83.9) | 55.2 (89.0) | 51.1 (82.4) | 3.2 (6.2) | -0.9 (-1.7) | 0.981 | 0.794 |
|  |  | (72.7—92.8) |  |  |  |  |  |  |
| II | 62 (12.0) | 57 (91.9) | 54.5 (87.9) | 47.1 (76.0) | -2.5 (-4.4) | -9.9 (-17.4) | 0.936 | 0.546 |
|  |  | (82.6—98.8) |  |  |  |  |  |  |
| III | 39 (7.5) | 36 (92.3) | 29.3 (75.1) | 20.2 (51.8) | -6.7 (-18.6) | -15.8 (-43.9) | 0.511 | 0.004 |
|  |  | (79.8—100.0) |  |  |  |  |  |  |
| Unknown | 14 (2.7) | 12 (85.7) | 11.5 (82.1) | 10.4 (74.3) | -0.5 (-4.2) | -1.6 (-13.3) | 0.987 | 0.782 |
|  |  | (64.7—100.0) |  |  |  |  |  |  |
| **HR status** |  |  |  |  |  |  |  |  |
| Positive | 354 (68.5) | 310 (87.6) | 308.0 (87.0) | 287.3 (81.2) | -2.0 (-0.6) | -22.7 (-7.3) | 0.988 | 0.779 |
|  |  | (81.6—89.6) |  |  |  |  |  |  |
| Negative | 163 (31.5) | 147 (90.2) | 138.0 (84.7) | 118.8 (72.9) | -9.0 (-6.1) | -28.2 (-19.2) | 0.580 | 0.002 |
|  |  | (82.9—93.8) |  |  |  |  |  |  |
| **ER Positive** |  |  |  |  |  |  |  |  |
| Positive | 340 (65.8) | 300 (88.2) | 295.6 (86.9) | 277.2 (81.5) | -4.4 (-1.5) | -22.8 (-7.6) | 0.982 | 0.855 |
|  |  | (82.3—90.2) |  |  |  |  |  |  |
| **ER Negative** |  |  |  |  |  |  |  |  |
| Positive | 14 (2.7) | 10 (71.4) | 12.4 (88.6) | 10.0 (71.4) | 2.4 (24.0) | 0.0 (0.0) | 0.839 | 0.743 |
|  |  | (51.3—99.5) |  |  |  |  |  |  |
| Negative | 163 (31.5) | 147 (90.2) | 138.0 (84.7) | 118.8 (72.9) | -9.0 (-6.1) | -28.2 (-19.2) | 0.580 | 0.002 |
|  |  | (82.9—93.8) |  |  |  |  |  |  |
| **Histology** |  |  |  |  |  |  |  |  |
| Ductal | 459 (88.8) | 406 (88.5) | 395.4 (86.1) | 358.8 (78.2) | -10.6 (-2.6) | -47.2 (-11.6) | 0.857 | 0.017 |
|  |  | (83.0—89.8) |  |  |  |  |  |  |
| Lobular | 16 (3.1) | 13 (81.2) | 14.3 (89.4) | 12.9 (80.6) | 1.3 (10.0) | -0.1 (-0.8) | 0.967 | 0.905 |
|  |  | (62.1—100.0) |  |  |  |  |  |  |
| Mixed ductal/lobular | 12 (2.3) | 12 (100.0) | 10.7 (89.2) | 9.8 (81.7) | -1.3 (-10.8) | -2.2 (-18.3) | 0.999 | 0.991 |
|  |  | (100.0—100.0) |  |  |  |  |  |  |
| Others | 30 (5.8) | 26 (86.7) | 25.7 (85.7) | 24.5 (81.7) | -0.3 (-1.2) | -1.5 (-5.8) | 0.989 | 0.934 |
|  |  | (73.9—99.7) |  |  |  |  |  |  |
| **ER Positive** |  |  |  |  |  |  |  |  |
| Ductal | 298 (57.6) | 261 (87.6) | 258.7 (86.8) | 242.6 (81.4) | -2.3 (-0.9) | -18.4 (-7.0) | 0.987 | 0.864 |
|  |  | (81.0—89.8) |  |  |  |  |  |  |
| Lobular | 14 (2.7) | 12 (85.7) | 12.5 (89.3) | 11.5 (82.1) | 0.5 (4.2) | -0.5 (-4.2) | 0.994 | 0.963 |
|  |  | (67.1—100.0) |  |  |  |  |  |  |
| Mixed ductal/lobular | 11 (2.1) | 11 (100.0) | 9.7 (88.2) | 9.0 (81.8) | -1.3 (-11.8) | -2.0 (-18.2) | 0.999 | 0.991 |
|  |  | (100.0—100.0) |  |  |  |  |  |  |
| Others | 17 (3.3) | 16 (94.1) | 14.8 (87.1) | 14.2 (83.5) | -1.2 (-7.5) | -1.8 (-11.3) | 1.000 | 1.000 |
|  |  | (82.6—100.0) |  |  |  |  |  |  |
| **ER Negative** |  |  |  |  |  |  |  |  |
| Ductal | 161 (31.1) | 145 (90.1) | 136.7 (84.9) | 116.2 (72.2) | -8.3 (-5.7) | -28.8 (-19.9) | 0.507 | 0.002 |
|  |  | (83.0—93.8) |  |  |  |  |  |  |
| Lobular | 2 (0.4) | 1 (50.0) | 1.8 (90.0) | 1.4 (70.0) | 0.8 (80.0) | 0.4 (40.0) | 0.342 | 0.466 |
|  |  | / |  |  |  |  |  |  |
| Mixed ductal/lobular | 1 (0.2) | 1 (100.0) | 1.0 (100.0) | 0.8 (80.0) | 0.0 (0.0) | -0.2 (-20.0) | NA | NA |
|  |  | / |  |  |  |  |  |  |
| Others | 13 (2.5) | 10 (76.9) | 10.9 (83.8) | 10.3 (79.2) | 0.9 (9.0) | 0.3 (3.0) | 0.956 | 0.631 |
|  |  | (54.9—100.0) |  |  |  |  |  |  |
| **Diagnosis year** |  |  |  |  |  |  |  |  |
| 2010-2012 | 517 (100.0) | 457 (88.4) | 446.0 (86.3) | 406.1 (78.5) | -11.0 (-2.4) | -50.9 (-11.1) | 0.860 | 0.023 |
|  |  | (83.2—89.6) |  |  |  |  |  |  |
| **ER Positive** |  |  |  |  |  |  |  |  |
| 2010-2012 | 340 (65.8) | 300 (88.2) | 295.6 (86.9) | 277.2 (81.5) | -4.4 (-1.5) | -22.8 (-7.6) | 0.982 | 0.855 |
|  |  | (82.3—90.2) |  |  |  |  |  |  |
| **ER Negative** |  |  |  |  |  |  |  |  |
| 2010-2012 | 177 (34.2) | 157 (88.7) | 150.4 (85.0) | 128.8 (72.8) | -6.6 (-4.2) | -28.2 (-18.0) | 0.522 | 0.002 |
|  |  | (81.3—92.3) |  |  |  |  |  |  |
| **Lymph or vascular invasion** | |  |  |  |  |  |  |  |
| Yes | 50 (9.7) | 43 (86.0) | 40.0 (80.0) | 31.9 (63.8) | -3.0 (-7.0) | -11.1 (-25.8) | 0.913 | 0.277 |
|  |  | (70.6—95.0) |  |  |  |  |  |  |
| No | 96 (18.6) | 91 (94.8) | 84.4 (87.9) | 77.7 (80.9) | -6.6 (-7.3) | -13.3 (-14.6) | 0.930 | 0.704 |
|  |  | (88.9—99.2) |  |  |  |  |  |  |
| Unknown | 371 (71.8) | 323 (87.1) | 321.6 (86.7) | 296.5 (79.9) | -1.4 (-0.4) | -26.5 (-8.2) | 0.945 | 0.136 |
|  |  | (81.0—88.9) |  |  |  |  |  |  |
| **ER Positive** |  |  |  |  |  |  |  |  |
| Yes | 34 (6.6) | 29 (85.3) | 27.6 (81.2) | 23.2 (68.2) | -1.4 (-4.8) | -5.8 (-20.0) | 0.998 | 0.944 |
|  |  | (67.3—97.5) |  |  |  |  |  |  |
| No | 63 (12.2) | 60 (95.2) | 56.4 (89.5) | 53.7 (85.2) | -3.6 (-6.0) | -6.3 (-10.5) | 0.998 | 1.000 |
|  |  | (88.7—100.0) |  |  |  |  |  |  |
| Unknown | 243 (47.0) | 211 (86.8) | 211.6 (87.1) | 200.3 (82.4) | 0.6 (0.3) | -10.7 (-5.1) | 0.958 | 0.966 |
|  |  | (79.8—89.7) |  |  |  |  |  |  |
| **ER Negative** |  |  |  |  |  |  |  |  |
| Yes | 16 (3.1) | 14 (87.5) | 12.4 (77.5) | 8.6 (53.8) | -1.6 (-11.4) | -5.4 (-38.6) | 0.760 | 0.298 |
|  |  | (65.7—100.0) |  |  |  |  |  |  |
| No | 33 (6.4) | 31 (93.9) | 28.0 (84.8) | 23.9 (72.4) | -3.0 (-9.7) | -7.1 (-22.9) | 0.862 | 0.589 |
|  |  | (83.5—100.0) |  |  |  |  |  |  |
| Unknown | 128 (24.8) | 112 (87.5) | 110.0 (85.9) | 96.3 (75.2) | -2.0 (-1.8) | -15.7 (-14.0) | 0.880 | 0.066 |
|  |  | (79.0—92.3) |  |  |  |  |  |  |
| **Anatomic neoplasm** | |  |  |  |  |  |  |  |
| Left | 268 (51.8) | 237 (88.4) | 231.3 (86.3) | 212.5 (79.3) | -5.7 (-2.4) | -24.5 (-10.3) | 0.915 | 0.289 |
|  |  | (82.2—91.0) |  |  |  |  |  |  |
| Right | 249 (48.2) | 220 (88.4) | 214.7 (86.2) | 193.6 (77.8) | -5.3 (-2.4) | -26.4 (-12.0) | 0.942 | 0.222 |
|  |  | (81.6—91.0) |  |  |  |  |  |  |
| **ER Positive** |  |  |  |  |  |  |  |  |
| Left | 191 (36.9) | 169 (88.5) | 165.2 (86.5) | 153.8 (80.5) | -3.8 (-2.2) | -15.2 (-9.0) | 0.979 | 0.839 |
|  |  | (81.5—92.0) |  |  |  |  |  |  |
| Right | 149 (28.8) | 131 (87.9) | 130.4 (87.5) | 123.4 (82.8) | -0.6 (-0.5) | -7.6 (-5.8) | 0.976 | 0.991 |
|  |  | (79.7—92.0) |  |  |  |  |  |  |
| **ER Negative** |  |  |  |  |  |  |  |  |
| Left | 77 (14.9) | 68 (88.3) | 66.1 (85.8) | 58.7 (76.2) | -1.9 (-2.8) | -9.3 (-13.7) | 0.705 | 0.194 |
|  |  | (78.2—95.0) |  |  |  |  |  |  |
| Right | 100 (19.3) | 89 (89.0) | 84.3 (84.3) | 70.1 (70.1) | -4.7 (-5.3) | -18.9 (-21.2) | 0.813 | 0.018 |
|  |  | (80.1—94.5) |  |  |  |  |  |  |
| Metastasis after diagnosis |  |  |  |  |  |  |  |  |
| Yes | 136 (26.3) | 95 (69.9) | 116.1 (85.4) | 104.8 (77.1) | 21.1 (22.2) | 9.8 (10.3) | 0.776 | 0.439 |
|  |  | (59.9—76.3) |  |  |  |  |  |  |
| No | 381 (73.7) | 362 (95.0) | 329.9 (86.6) | 301.3 (79.1) | -32.1 (-8.9) | -60.7 (-16.8) | 0.916 | 0.061 |
|  |  | (91.3—96.6) |  |  |  |  |  |  |
| **ER Positive** |  |  |  |  |  |  |  |  |
| Yes | 93 (18.0) | 66 (71.0) | 79.4 (85.4) | 74.0 (79.6) | 13.4 (20.3) | 8.0 (12.1) | 0.866 | 0.978 |
|  |  | (59.8—79.4) |  |  |  |  |  |  |
| No | 247 (47.8) | 234 (94.7) | 216.3 (87.6) | 203.3 (82.3) | -17.7 (-7.6) | -30.7 (-13.1) | 0.998 | 0.914 |
|  |  | (90.2—97.0) |  |  |  |  |  |  |
| **ER Negative** |  |  |  |  |  |  |  |  |
| Yes | 43 (8.3) | 29 (67.4) | 36.7 (85.3) | 30.8 (71.6) | 7.7 (26.6) | 1.8 (6.2) | 0.728 | 0.104 |
|  |  | (51.6—81.6) |  |  |  |  |  |  |
| No | 134 (25.9) | 128 (95.5) | 113.7 (84.9) | 98.0 (73.1) | -14.3 (-11.2) | -30.0 (-23.4) | 0.781 | 0.035 |
|  |  | (90.3—98.9) |  |  |  |  |  |  |
| Liver Metastasis after diagnosis |  |  |  |  |  |  |  |  |
| Yes | 27 (5.2) | 9 (33.3) | 23.1 (85.6) | 20.0 (74.1) | 14.1 (156.7) | 11.0 (122.2) | 0.354 | 0.522 |
|  |  | (14.9—53.3) |  |  |  |  |  |  |
| No | 490 (94.8) | 448 (91.4) | 422.9 (86.3) | 386.1 (78.8) | -25.1 (-5.6) | -61.9 (-13.8) | 0.861 | 0.029 |
|  |  | (86.9—92.8) |  |  |  |  |  |  |
| **ER Positive** |  |  |  |  |  |  |  |  |
| Yes | 17 (3.3) | 7 (41.2) | 14.4 (84.7) | 13.0 (76.5) | 7.4 (105.7) | 6.0 (85.7) | 0.543 | 0.659 |
|  |  | (15.5—69.3) |  |  |  |  |  |  |
| No | 323 (62.5) | 293 (90.7) | 281.2 (87.1) | 264.3 (81.8) | -11.8 (-4.0) | -28.7 (-9.8) | 0.997 | 0.827 |
|  |  | (85.4—92.8) |  |  |  |  |  |  |
| **ER Negative** |  |  |  |  |  |  |  |  |
| Yes | 10 (1.9) | 2 (20.0) | 8.7 (87.0) | 7.1 (71.0) | 6.7 (335.0) | 5.1 (255.0) | 0.499 | 0.195 |
|  |  | (5.8—69.1) |  |  |  |  |  |  |
| No | 167 (32.3) | 155 (92.8) | 141.7 (84.9) | 121.8 (72.9) | -13.3 (-8.6) | -33.2 (-21.4) | 0.559 | 0.003 |
|  |  | (86.8—96.2) |  |  |  |  |  |  |
| Bone Metastasis after diagnosis |  |  |  |  |  |  |  |  |
| Yes | 89 (17.2) | 58 (65.2) | 76.5 (86.0) | 70.0 (78.7) | 18.5 (31.9) | 12.0 (20.7) | 0.675 | 0.759 |
|  |  | (53.9—74.6) |  |  |  |  |  |  |
| No | 428 (82.8) | 399 (93.2) | 369.5 (86.3) | 336.1 (78.5) | -29.5 (-7.4) | -62.9 (-15.8) | 0.937 | 0.042 |
|  |  | (88.9—94.7) |  |  |  |  |  |  |
| **ER Positive** |  |  |  |  |  |  |  |  |
| Yes | 63 (12.2) | 43 (68.3) | 53.6 (85.1) | 50.0 (79.4) | 10.6 (24.7) | 7.0 (16.3) | 0.750 | 0.913 |
|  |  | (56.1—80.0) |  |  |  |  |  |  |
| No | 277 (53.6) | 257 (92.8) | 242.0 (87.4) | 227.2 (82.0) | -15.0 (-5.8) | -29.8 (-11.6) | 1.000 | 0.947 |
|  |  | (87.6—95.0) |  |  |  |  |  |  |
| **ER Negative** |  |  |  |  |  |  |  |  |
| Yes | 26 (5.0) | 15 (57.7) | 22.9 (88.1) | 19.9 (76.5) | 7.9 (52.7) | 4.9 (32.7) | 0.688 | 0.527 |
|  |  | (37.7—78.4) |  |  |  |  |  |  |
| No | 151 (29.2) | 142 (94.0) | 127.5 (84.4) | 108.9 (72.1) | -14.5 (-10.2) | -33.1 (-23.3) | 0.719 | 0.009 |
|  |  | (88.4—97.4) |  |  |  |  |  |  |
| Brain Metastasis after diagnosis |  |  |  |  |  |  |  |  |
| Yes | 23 (4.4) | 15 (65.2) | 18.9 (82.2) | 16.7 (72.6) | 3.9 (26.0) | 1.7 (11.3) | 0.881 | 0.717 |
|  |  | (42.0—85.8) |  |  |  |  |  |  |
| No | 494 (95.6) | 442 (89.5) | 427.1 (86.5) | 389.4 (78.8) | -14.9 (-3.4) | -52.6 (-11.9) | 0.777 | 0.021 |
|  |  | (84.5—90.8) |  |  |  |  |  |  |
| **ER Positive** |  |  |  |  |  |  |  |  |
| Yes | 19 (3.7) | 12 (63.2) | 15.9 (83.7) | 14.6 (76.8) | 3.9 (32.5) | 2.6 (21.7) | 0.959 | 0.515 |
|  |  | (42.3—88.3) |  |  |  |  |  |  |
| No | 321 (62.1) | 288 (89.7) | 279.7 (87.1) | 262.6 (81.8) | -8.3 (-2.9) | -25.4 (-8.8) | 0.968 | 0.780 |
|  |  | (84.0—91.8) |  |  |  |  |  |  |
| **ER Negative** |  |  |  |  |  |  |  |  |
| Yes | 4 (0.8) | 3 (75.0) | 3.0 (75.0) | 2.1 (52.5) | 0.0 (0.0) | -0.9 (-30.0) | 0.613 | 0.325 |
|  |  | (12.5—100.0) |  |  |  |  |  |  |
| No | 173 (33.5) | 154 (89.0) | 147.4 (85.2) | 126.8 (73.3) | -6.6 (-4.3) | -27.2 (-17.7) | 0.559 | 0.002 |
|  |  | (81.9—92.7) |  |  |  |  |  |  |
| Lung Metastasis after diagnosis |  |  |  |  |  |  |  |  |
| Yes | 40 (7.7) | 18 (45.0) | 35.5 (88.8) | 31.8 (79.5) | 17.5 (97.2) | 13.8 (76.7) | 0.482 | 0.525 |
|  |  | (29.2—61.5) |  |  |  |  |  |  |
| No | 477 (92.3) | 439 (92.0) | 410.6 (86.1) | 374.3 (78.5) | -28.4 (-6.5) | -64.7 (-14.7) | 0.916 | 0.051 |
|  |  | (87.7—93.4) |  |  |  |  |  |  |
| **ER Positive** |  |  |  |  |  |  |  |  |
| Yes | 22 (4.3) | 10 (45.5) | 19.7 (89.5) | 18.4 (83.6) | 9.7 (97.0) | 8.4 (84.0) | 0.852 | 0.804 |
|  |  | (27.8—71.6) |  |  |  |  |  |  |
| No | 318 (61.5) | 290 (91.2) | 275.9 (86.8) | 258.9 (81.4) | -14.1 (-4.9) | -31.1 (-10.7) | 0.991 | 0.893 |
|  |  | (86.0—93.3) |  |  |  |  |  |  |
| **ER Negative** |  |  |  |  |  |  |  |  |
| Yes | 18 (3.5) | 8 (44.4) | 15.8 (87.8) | 13.4 (74.4) | 7.8 (97.5) | 5.4 (67.5) | 0.318 | 0.270 |
|  |  | (21.5—72.5) |  |  |  |  |  |  |
| No | 159 (30.8) | 149 (93.7) | 134.6 (84.7) | 115.4 (72.6) | -14.4 (-9.7) | -33.6 (-22.6) | 0.525 | 0.007 |
|  |  | (88.1—97.1) |  |  |  |  |  |  |
| **Type of surgery** |  |  |  |  |  |  |  |  |
| Mastectomy | 395 (76.4) | 346 (87.6) | 335.8 (85.0) | 302.9 (76.7) | -10.2 (-2.9) | -43.1 (-12.5) | 0.741 | 0.011 |
|  |  | (81.1—88.9) |  |  |  |  |  |  |
| Breast-conserving | 121 (23.4) | 111 (91.7) | 109.6 (90.6) | 102.6 (84.8) | -1.4 (-1.3) | -8.4 (-7.6) | 0.994 | 0.991 |
|  |  | (85.9—96.5) |  |  |  |  |  |  |
| **ER Positive** |  |  |  |  |  |  |  |  |
| Mastectomy | 256 (49.5) | 222 (86.7) | 219.9 (85.9) | 205.1 (80.1) | -2.1 (-0.9) | -16.9 (-7.6) | 0.952 | 0.832 |
|  |  | (79.0—89.0) |  |  |  |  |  |  |
| Breast-conserving | 83 (16.1) | 78 (94.0) | 75.1 (90.5) | 71.6 (86.3) | -2.9 (-3.7) | -6.4 (-8.2) | 0.996 | 0.997 |
|  |  | (88.2—99.2) |  |  |  |  |  |  |
| **ER Negative** |  |  |  |  |  |  |  |  |
| Mastectomy | 139 (26.9) | 124 (89.2) | 115.9 (83.4) | 97.9 (70.4) | -8.1 (-6.5) | -26.1 (-21.0) | 0.478 | 0.002 |
|  |  | (80.9—93.3) |  |  |  |  |  |  |
| Breast-conserving | 38 (7.4) | 33 (86.8) | 34.5 (90.8) | 31.0 (81.6) | 1.5 (4.5) | -2.0 (-6.1) | 0.999 | 0.995 |
|  |  | (74.9—98.2) |  |  |  |  |  |  |
| **Lymph node procedure** | |  |  |  |  |  |  |  |
| SLNB | 100 (19.3) | 89 (89.0) | 89.9 (89.9) | 84.0 (84.0) | 0.9 (1.0) | -5.0 (-5.6) | 0.993 | 0.961 |
|  |  | (81.3—94.8) |  |  |  |  |  |  |
| ALND | 286 (55.3) | 253 (88.5) | 243.6 (85.2) | 220.0 (76.9) | -9.4 (-3.7) | -33.0 (-13.0) | 0.829 | 0.055 |
|  |  | (81.4—90.4) |  |  |  |  |  |  |
| SLNB+ALND | 68 (13.2) | 62 (91.2) | 59.8 (87.9) | 55.0 (80.9) | -2.2 (-3.5) | -7.0 (-11.3) | 0.845 | 0.841 |
|  |  | (83.3—98.0) |  |  |  |  |  |  |
| Unknown | 63 (12.2) | 53 (84.1) | 52.8 (83.8) | 47.0 (74.6) | -0.2 (-0.4) | -6.0 (-11.3) | 0.959 | 0.510 |
|  |  | (71.7—92.5) |  |  |  |  |  |  |
| **ER Positive** |  |  |  |  |  |  |  |  |
| SLNB | 72 (13.9) | 63 (87.5) | 64.1 (89.0) | 61.2 (85.0) | 1.1 (1.7) | -1.8 (-2.9) | 0.994 | 0.951 |
|  |  | (78.0—94.9) |  |  |  |  |  |  |
| ALND | 184 (35.6) | 162 (88.0) | 158.4 (86.1) | 147.1 (79.9) | -3.6 (-2.2) | -14.9 (-9.2) | 0.988 | 0.947 |
|  |  | (79.8—91.2) |  |  |  |  |  |  |
| SLNB+ALND | 46 (8.9) | 44 (95.7) | 40.7 (88.5) | 38.5 (83.7) | -3.3 (-7.5) | -5.5 (-12.5) | 0.957 | 0.952 |
|  |  | (89.0—100.0) |  |  |  |  |  |  |
| Unknown | 38 (7.4) | 31 (81.6) | 32.5 (85.5) | 30.4 (80.0) | 1.5 (4.8) | -0.6 (-1.9) | 0.811 | 0.993 |
|  |  | (66.0—94.1) |  |  |  |  |  |  |
| **ER Negative** |  |  |  |  |  |  |  |  |
| SLNB | 28 (5.4) | 26 (92.9) | 25.8 (92.1) | 22.8 (81.4) | -0.2 (-0.8) | -3.2 (-12.3) | 0.960 | 0.899 |
|  |  | (83.2—100.0) |  |  |  |  |  |  |
| ALND | 102 (19.7) | 91 (89.2) | 85.3 (83.6) | 72.9 (71.5) | -5.7 (-6.3) | -18.1 (-19.9) | 0.651 | 0.019 |
|  |  | (79.8—94.4) |  |  |  |  |  |  |
| SLNB+ALND | 22 (4.3) | 18 (81.8) | 19.1 (86.8) | 16.6 (75.5) | 1.1 (6.1) | -1.4 (-7.8) | 0.814 | 0.734 |
|  |  | (64.3—99.6) |  |  |  |  |  |  |
| Unknown | 25 (4.8) | 22 (88.0) | 20.3 (81.2) | 16.6 (66.4) | -1.7 (-7.7) | -5.4 (-24.5) | 0.993 | 0.251 |
|  |  | (72.0—100.0) |  |  |  |  |  |  |
| **Hormone Therapy** | |  |  |  |  |  |  |  |
| Yes | 327 (63.2) | 290 (88.7) | 286.2 (87.5) | 268.1 (82.0) | -3.8 (-1.3) | -21.9 (-7.6) | 0.985 | 0.929 |
|  |  | (83.4—91.1) |  |  |  |  |  |  |
| No | 190 (36.8) | 167 (87.9) | 159.8 (84.1) | 138.0 (72.6) | -7.2 (-4.3) | -29.0 (-17.4) | 0.682 | <0.001 |
|  |  | (79.0—90.6) |  |  |  |  |  |  |
| **AI(Aromatase Inhibitors)** | |  |  |  |  |  |  |  |
| Yes | 198 (38.3) | 171 (86.4) | 169.3 (85.5) | 160.4 (81.0) | -1.7 (-1.0) | -10.6 (-6.2) | 0.929 | 0.956 |
|  |  | (80.2—90.5) |  |  |  |  |  |  |
| **SERM(Selective Estrogen Receptor Modulators)** | | |  |  |  |  |  |  |
| Yes | 224 (43.3) | 200 (89.3) | 202.5 (90.4) | 188.0 (83.9) | 2.5 (1.2) | -12.0 (-6.0) | 0.987 | 0.958 |
|  |  | (83.5—92.6) |  |  |  |  |  |  |
| **Generation chemotherapy** | |  |  |  |  |  |  |  |
| No chemotherapy | 67 (13.0) | 52 (77.6) | 49.9 (74.5) | 45.8 (68.4) | -2.1 (-4.0) | -6.2 (-11.9) | 0.060 | 0.002 |
|  |  | (61.0—85.2) |  |  |  |  |  |  |
| Generation 2 | 81 (15.7) | 78 (96.3) | 72.7 (89.8) | 69.0 (85.2) | -5.3 (-6.8) | -9.0 (-11.5) | 1.000 | 0.971 |
|  |  | (90.7—100.0) |  |  |  |  |  |  |
| Generation 3 | 369 (71.4) | 327 (88.6) | 323.4 (87.6) | 291.3 (78.9) | -3.6 (-1.1) | -35.7 (-10.9) | 0.824 | 0.037 |
|  |  | (83.1—90.6) |  |  |  |  |  |  |
| **ER Positive** |  |  |  |  |  |  |  |  |
| No chemotherapy | 46 (8.9) | 36 (78.3) | 35.4 (77.0) | 34.1 (74.1) | -0.6 (-1.7) | -1.9 (-5.3) | 0.978 | 0.828 |
|  |  | (59.4—88.6) |  |  |  |  |  |  |
| Generation 2 | 59 (11.4) | 57 (96.6) | 53.9 (91.4) | 51.9 (88.0) | -3.1 (-5.4) | -5.1 (-8.9) | 1.000 | 1.000 |
|  |  | (90.9—100.0) |  |  |  |  |  |  |
| Generation 3 | 235 (45.5) | 207 (88.1) | 206.4 (87.8) | 191.3 (81.4) | -0.6 (-0.3) | -15.7 (-7.6) | 0.969 | 0.781 |
|  |  | (81.5—91.0) |  |  |  |  |  |  |
| **ER Negative** |  |  |  |  |  |  |  |  |
| No chemotherapy | 21 (4.1) | 16 (76.2) | 14.6 (69.5) | 11.7 (55.7) | -1.4 (-8.8) | -4.3 (-26.9) | 0.092 | 0.003 |
|  |  | (51.9—95.9) |  |  |  |  |  |  |
| Generation 2 | 22 (4.3) | 21 (95.5) | 18.8 (85.5) | 17.1 (77.7) | -2.2 (-10.5) | -3.9 (-18.6) | 0.985 | 0.969 |
|  |  | (83.6—100.0) |  |  |  |  |  |  |
| Generation 3 | 134 (25.9) | 120 (89.6) | 117.1 (87.4) | 100.0 (74.6) | -2.9 (-2.4) | -20.0 (-16.7) | 0.790 | 0.042 |
|  |  | (82.1—94.0) |  |  |  |  |  |  |
| **Targeted Molecular therapy** | |  |  |  |  |  |  |  |
| Yes | 46 (8.9) | 41 (89.1) | 41.0 (89.1) | 35.9 (78.0) | 0.0 (0.0) | -5.1 (-12.4) | 0.976 | 0.578 |
|  |  | (80.5—98.6) |  |  |  |  |  |  |
| No | 471 (91.1) | 416 (88.3) | 405.0 (86.0) | 370.2 (78.6) | -11.0 (-2.6) | -45.8 (-11.0) | 0.899 | 0.047 |
|  |  | (82.6—89.5) |  |  |  |  |  |  |
| **ER Positive** |  |  |  |  |  |  |  |  |
| Yes | 24 (4.6) | 21 (87.5) | 21.4 (89.2) | 19.4 (80.8) | 0.4 (1.9) | -1.6 (-7.6) | 0.947 | 0.962 |
|  |  | (75.2—100.0) |  |  |  |  |  |  |
| No | 316 (61.1) | 279 (88.3) | 274.3 (86.8) | 257.9 (81.6) | -4.7 (-1.7) | -21.1 (-7.6) | 0.970 | 0.883 |
|  |  | (82.0—90.3) |  |  |  |  |  |  |
| **ER Negative** |  |  |  |  |  |  |  |  |
| Yes | 22 (4.3) | 20 (90.9) | 19.6 (89.1) | 16.5 (75.0) | -0.4 (-2.0) | -3.5 (-17.5) | 0.991 | 0.603 |
|  |  | (79.7—100.0) |  |  |  |  |  |  |
| No | 155 (30.0) | 137 (88.4) | 130.8 (84.4) | 112.3 (72.5) | -6.2 (-4.5) | -24.7 (-18.0) | 0.538 | 0.006 |
|  |  | (80.1—92.2) |  |  |  |  |  |  |
| **Radiation therapy** | |  |  |  |  |  |  |  |
| Yes | 156 (30.2) | 135 (86.5) | 138.6 (88.8) | 121.3 (77.8) | 3.6 (2.7) | -13.7 (-10.1) | 0.959 | 0.215 |
|  |  | (79.6—91.3) |  |  |  |  |  |  |
| No | 361 (69.8) | 322 (89.2) | 307.4 (85.2) | 284.8 (78.9) | -14.6 (-4.5) | -37.2 (-11.6) | 0.933 | 0.218 |
|  |  | (83.1—90.8) |  |  |  |  |  |  |
| **ER Positive** |  |  |  |  |  |  |  |  |
| Yes | 100 (19.3) | 88 (88.0) | 89.0 (89.0) | 80.6 (80.6) | 1.0 (1.1) | -7.4 (-8.4) | 0.992 | 0.699 |
|  |  | (80.0—94.0) |  |  |  |  |  |  |
| No | 240 (46.4) | 212 (88.3) | 206.7 (86.1) | 196.6 (81.9) | -5.3 (-2.5) | -15.4 (-7.3) | 0.959 | 0.970 |
|  |  | (81.2—90.9) |  |  |  |  |  |  |
| **ER Negative** |  |  |  |  |  |  |  |  |
| Yes | 56 (10.8) | 47 (83.9) | 49.7 (88.8) | 40.7 (72.7) | 2.7 (5.7) | -6.3 (-13.4) | 0.986 | 0.395 |
|  |  | (73.3—93.7) |  |  |  |  |  |  |
| No | 121 (23.4) | 110 (90.9) | 100.7 (83.2) | 88.2 (72.9) | -9.3 (-8.5) | -21.8 (-19.8) | 0.556 | 0.010 |
|  |  | (82.6—95.2) |  |  |  |  |  |  |

Abbreviations: N = total number, CI = confidence interval, ER = oestrogen receptor, PR = progesterone receptor, HR = Hormone Receptor, HER2 = human epidermal growth factor receptor 2.

*The p-value was calculated by using a Chi squared-test. p-values indicated in bold are considered as statistically significant (p < 0.05). >5% difference was considered as clinically relevant.
